# Human neuronal signaling and communication assays to assess functional neurotoxicity

**DOI:** 10.1007/s00204-020-02956-3

**Published:** 2020-12-02

**Authors:** Dominik Loser, Jasmin Schaefer, Timm Danker, Clemens Möller, Markus Brüll, Ilinca Suciu, Anna-Katharina Ückert, Stefanie Klima, Marcel Leist, Udo Kraushaar

**Affiliations:** 1 NMI Natural and Medical Sciences Institute at the University of Tuebingen, 72770 Reutlingen, Germany; 2 NMI TT GmbH, 72770 Reutlingen, Germany; 3 In Vitro Toxicology and Biomedicine, Department Inaugurated by the Doerenkamp-Zbinden Foundation, University of Konstanz, Universitaetsstr. 10, 78457 Constance, Germany; 4 Life Sciences Faculty, Albstadt-Sigmaringen University, 72488 Sigmaringen, Germany

**Keywords:** Channel toxins, Neuronal network, Purinoceptor, Dopamine transporter, Network oscillations

## Abstract

Prediction of drug toxicity on the human nervous system still relies mainly on animal experiments. Here, we developed an alternative system allowing assessment of complex signaling in both individual human neurons and on the network level. The LUHMES cultures used for our approach can be cultured in 384-well plates with high reproducibility. We established here high-throughput quantification of free intracellular Ca^2+^ concentrations [Ca^2+^]_i_ as broadly applicable surrogate of neuronal activity and verified the main processes by patch clamp recordings. Initially, we characterized the expression pattern of many neuronal signaling components and selected the purinergic receptors to demonstrate the applicability of the [Ca^2+^]_i_ signals for quantitative characterization of agonist and antagonist responses on classical ionotropic neurotransmitter receptors. This included receptor sub-typing and the characterization of the anti-parasitic drug suramin as modulator of the cellular response to ATP. To exemplify potential studies on ion channels, we characterized voltage-gated sodium channels and their inhibition by tetrodotoxin, saxitoxin and lidocaine, as well as their opening by the plant alkaloid veratridine and the food-relevant marine biotoxin ciguatoxin. Even broader applicability of [Ca^2+^]_i_ quantification as an end point was demonstrated by measurements of dopamine transporter activity based on the membrane potential-changing activity of this neurotransmitter carrier. The substrates dopamine or amphetamine triggered [Ca^2+^]_i_ oscillations that were synchronized over the entire culture dish. We identified compounds that modified these oscillations by interfering with various ion channels. Thus, this new test system allows multiple types of neuronal signaling, within and between cells, to be assessed, quantified and characterized for their potential disturbance.

**Electronic supplementary material:**

The online version of this article (10.1007/s00204-020-02956-3) contains supplementary material, which is available to authorized users.

## Introduction

Assessment of adverse effects on the nervous system is still a challenge for the development of drugs and for many chemicals in other industry sectors (Schmidt et al. 2017; Walker et al. 2018). It is widely accepted that misleading outputs from traditional preclinical screenings contribute to the current decline in new drug applications, and therefore, more and better tests are required. The most frequent drug side effects observed after drug marketing are related to the disturbance of nervous system function (Redfern et al. 2010). In this context, it is important to note that the toxicity of excitable tissues like the nervous system (or the heart) differs from typical toxic effects observed, e.g., in the liver. For electrically active cells, pronounced impairment can occur in the absence of any morphological changes. The undetected toxicity of drugs is still a major cause of death in the EU (Giardina et al. 2018) and the USA (Sonawane et al. 2018). Neurotoxicity and cardiotoxicity account together for nearly 60% of drug rejections during trials or post-commercialization (McNaughton et al. 2014; Onakpoya et al. 2016; Walker et al. 2018). In the case of the nervous system, functional toxicants may lead to sensory disturbances, nausea, cognitive impairment or seizures. The occurrence of such adverse effects in man is not predicted well by classical animal models (Olson et al. 2000; Mead et al. 2016), and a large consortium of pharmaceutical industry has therefore initiated the NeuroDeRisk project within the innovative medicines initiative 2 (IMI2) of the Horizon2020 framework program (https://cordis.europa.eu/project/id/821528).

Several assays have been developed that assess the capacity of test compounds to kill neurons or to affect their morphology (Forsby et al. 2009; Wilson et al. 2014; Barbosa et al. 2015; Schultz et al. 2015). Some of them have proven useful also for larger screens, or were optimized to detect specific cell damage, e.g., to mitochondria (Delp et al. 2018a, 2019). However, such assays fail to detect several functional toxicants. It is therefore important that also neurophysiological end points can be robustly assessed. One approach is to measure the effects on a large panel of known receptors, enzymes, transporters and channels that are required for neuronal function (Pottel et al. 2020). A more economic variant of this approach uses a physiological parameter that is easily measurable and relates to many of the above toxicant targets. The change of the free intracellular Ca^2+^ concentration [Ca^2+^]_i_ is such an end point. The quantification can be performed at high throughput by using live-cell fluorescence imaging of neuronal cultures loaded with calcium-sensitive dyes (Sirenko et al. 2019; Grunwald et al. 2019; Karreman et al. 2020; Brüll et al. 2020).

Two major issues have to be addressed for establishment of an assay on this basis. First, a test system is required that is sufficiently robust to allow comparisons from cell to cell, from well to well, from plate to plate and also between biological replicates (= different cell preparations/assay days). Second, neuronal network features need to be captured. Testing of individual cells alone does not fully capture the neuronal physiology. It can assess many toxicant targets, but not the coupling of neurons with one another (the major functional feature of the nervous system).

Current attempts to develop improved neurotoxicity assays tackle these two issues in different ways (Schultz et al. 2015). The main options for test systems are rat primary neurons (Forsby et al. 2009; Sandström et al. 2017; Bradley et al. 2018; Kreir et al. 2018; Millard et al. 2019), cells differentiated from pluripotent stem cells (Pei et al. 2016; Sherman and Bang 2018; Sirenko et al. 2019; Tukker et al. 2020; Brüll et al. 2020), and cell lines (Forsby et al. 2009; Krug et al. 2013; Stiegler et al. 2011; Klima et al. 2020). The latter often have the disadvantage that they do not form effective synapses. Neurons derived from iPSC have a large potential, as shown by some screen applications (Xu et al. 2013; Ryan et al. 2016; Pei et al. 2016; Brownjohn et al. 2017; Kondo et al. 2017; Sherman and Bang 2018; Sirenko et al. 2019; Tukker et al. 2020), but their maturity and reproducibility are hard to control (Handel et al. 2016; Xia et al. 2016; Volpato et al. 2018; Little et al. 2019; Volpato and Webber 2020), and costs are very high (McKernan and Watt 2013; Bravery 2015; Engle et al. 2018; Huang et al. 2019). Primary cells can form excellent networks and contain many cell types of interest. They have for a long time been the major model used for mechanistic studies (Hansson et al. 2000; Gerhardt et al. 2001). However, they are usually of rodent origin, and molecular epitopes, gene expression programs and physiological functions may differ between species (Leist and Hartung 2013).

Several approaches have been developed to test for neuronal connectivity. For instance, paired patch clamp recordings of synaptically connected pre- and post-synaptic cells allow the investigation of neuronal transmission within a local network (Kraushaar and Jonas 2000; Hefft et al. 2002). An approach that allows more throughput are microelectrode arrays (MEA) that record extracellular field potentials of spontaneously active neuronal networks (Hogberg et al. 2011; McConnell et al. 2012; Nicolas et al. 2014; Alloisio et al. 2015; Odawara et al. 2016; Vassallo et al. 2016; Kraushaar et al. 2017; Bader et al. 2017; Bradley et al. 2018; Kreir et al. 2018; Tukker et al. 2018, 2020). By comparison of the firing patterns measured on various electrodes in a culture dish, network synchronization parameters can be derived. This system can thus assess toxicant effects both on the (averaged) function of individual neurons (e.g., spiking activity) or on network activity and allows therefore comprehensive screens (Alloisio et al. 2015; Kraushaar et al. 2017; Bader et al. 2017; Bradley et al. 2018; Kreir et al. 2018; Tukker et al. 2018, 2020). The use of rat neurons is most established for such MEA assays, but also human iPSC neurons are increasingly being used (Odawara et al. 2016, 2018; Kraushaar et al. 2017; Tukker et al. 2018, 2020). To this date, the cost and complexity of the latter approach have prevented larger screens, and only few studies requiring at least medium throughput (concentration–response curves with several replicates and assay conditions) have been published (Odawara et al. 2016, 2018; Kraushaar et al. 2017; Tukker et al. 2020).

We therefore investigated new strategies that will lead to an in vitro neurotoxicity assay using human cells, allowing for highly reproducible assay conditions at low cost and permitting measurements on single cell function as well as on network properties. As test system basis, we used LUHMES cells, which are conditionally immortalized, but non-transformed (Gutbier et al. 2018) human neurons that are well established for morphological, metabolical and biochemical neurotoxicity testing (Krug et al. 2013, 2014; Zhang et al. 2014; Lohren et al. 2015; Smirnova et al. 2016; Harris et al. 2017; Tong et al. 2017; Witt et al. 2017; Delp et al. 2018a, b, 2019; Brüll et al. 2020). We established Ca^2+^-signaling as the main end point, on the population level, as well as on the level of individual cells and confirmed their basic neuroexcitability parameters. Examples are provided for the assessment of toxicants affecting ion channels, receptors and transporters with high reproducibility and data accuracy. Finally, oscillations of [Ca^2+^]_i_ across the entire culture dish were identified and exemplified as readout for neuronal connectivity and as a measure to identify compounds modifying neuronal network features.

## Materials and methods

### Materials and chemicals

An overview of experimental tool compounds and toxicants is given in table S1.

Pacific ciguatoxin (pCTX) isolated from a moray eel was provided by the laboratory of Richard Lewis, University of Queensland, Brisbane, Australia.

### Cell culture

The cultivation of the LUHMES cells was performed as described earlier (Scholz et al. 2011; Krug et al. 2013; Schildknecht et al. 2013). In brief, LUHMES cells were cultured in standard cell culture flasks (Sarstedt) that were pre-coated with 50 µg/ml poly-l-ornithine (PLO) and 1 µg/ml fibronectin (Sigma-Aldrich) in H_2_O overnight at 37 °C. The cells were maintained in proliferation medium containing advanced DMEM/F12 (Gibco) with 2 mM L-glutamine (Sigma Aldrich), 1 × N2-supplement (Gibco) and 40 ng/ml recombinant human basic fibroblast growth factor (FGF-2, R&D Systems). The cells were kept at 37 °C and 5% CO_2_ and passaged three times a week, when the culture reached a confluency of 75–90%. Cells were used until passage 18. For differentiation, cells were cultured in differentiation medium consisting of advanced DMEM/F12 (Gibco) supplemented with 2 mM L-glutamine (Sigma Aldrich), 1 × N2-supplement (Gibco), 1 mM N6,2′-0-dibutyryl 3′,5′-cyclic adenosine monophosphate (cAMP) (Sigma Aldrich), 1 µg/ml tetracycline (Sigma Aldrich) and 2 ng/ml recombinant human glial cell-derived neurotrophic factor (GDNF, R&D Systems).

For automated patch clamp recordings, the cells were differentiated for 9 days. The medium was changed every other day, supplemented with 1 µg/ml laminin (Sigma Aldrich). For Ca^2+^-imaging, the cells were pre-differentiated for 48 h in cell culture flasks, detached and plated at a density of 20.000 cells per well on 0.1% PEI-coated 384-well plates (Greiner Bio-One), respectively. For manual patch clamp recordings, the cells were plated at a density of 750 cells/µl on 0.1% PEI-coated glass coverslips. The cells were further differentiated for another 7–8 days. 50% of the medium was exchanged every 2–3 days.

### Dopamine uptake

LUHMES cells were differentiated for 6 days in a 24-well format and then treated with the indicated DAT inhibitors. After 15 min, the natural DAT substrate dopamine (DA, 10 µM) was added in radioactively-labeled (^3^H, 1.5 Bq/mol) form. After 10 min at 37 °C, the supernatant was removed and cells were washed with PBS. Then lysis buffer was added (PBS containing 0.2% Triton X-100). The amount of radioactive label taken up by the cells as well as the residual activity in the cell supernatant (plus washing buffer) was measured on a scintillation counter. All inhibitor data were normalized to the uptake of cells only exposed to solvent (1.2 ± 0.4 nmol DA/10^6^ cells).

### Gene expression profiling

Five biological replicates were generated from LUHMES cells differentiated for 2, 3, 5, 6, 8, 10, and 11 days, as well as from undifferentiated LUHMES cells (day 0). These samples were prepared for transcriptome analysis considering genes of neurotransmitter receptors, ion channels, and calcium binding proteins. Samples were analyzed via the TempO-Seq assay, which is a targeted RNA sequencing method developed by BioSpyder Technologies Inc. (Carlsbad, CA, USA). The method is described in detail in House et al. (2017). For sample preparation, LUHMES grown in 96-well plates were lysed in 25 µl 1 × BioSpyder lysis buffer according to the manufacturer’s instructions. The lysate from ten wells was pooled for each sample. Samples were stored at − 80 °C before shipping on dry ice to BioClavis (BioClavis, ltd., Glasgow, UK) for TempO-Seq analysis. The resulting FASTQ files were aligned using the STAR algorithm to a pseudo-transcriptome by BioClavis and eventually normalized and standardized to a data format of x gene specific counts per one million reads. Traditional whole genome RNA sequencing (RNAseq) was performed for comparison and validation. Cells were cultured in six-well plates. For sample preparation, the medium was removed and cells were lysed in TriFast reagent (Peqlab, VWR, USA). The lysate of six wells was pooled for each sample. Samples were stored at − 20 °C until they were sent on dry ice to the department of toxicogenomics at the University Maastricht, The Netherlands, for RNAseq analysis.

### ***Ca***^***2***+^-imaging

Ca^2+^-imaging was performed using HT Functional Drug Screening System FDSS/µCELL (Hamamatsu Photonics) at nominal 37 °C. The FDSS/µCell system enables the indirect recording of changes of intracellular Ca^2+^ [Ca^2+^]_i_ via a Ca^2+^-sensitive fluorescent dye. The fluorescence signal of a complete 384-well plate is acquired at once with a high-speed and high-sensitivity digital ImagEM X2 EM-CCD camera (Electron Multiplying Charge-Coupled Device, Hamamatsu Photonics), but with limited spatial resolution. Therefore, the software only determines the mean fluorescence signal of each well. The signals of individual cells could not be captured. For compound application, the integrated dispenser head with 384 pipette tips was used, which can add the test compound to all wells simultaneously. The cells were preincubated with Cal-520 AM (AAT Bioquest) at a concentration of 1 µM for 1 h at 37 °C. For recording, the medium was exchanged by a buffer solution containing [mM]: 135 NaCl, 5 KCl, 0.2 MgCl_2_, 2.5 CaCl_2_, 10 HEPES and 10 D-glucose, pH 7.4. Test compound application was executed after obtaining a 1.5 min baseline recording. Where applicable, a second application was executed 4.5 min after the first application. The total recording never exceeded 8 min.

### Automated patch clamp recordings

Automated patch clamp recordings were performed on a QPatch (Sophion Bioscience) with 16X QPlates. After detachment, the cells were resuspended at a concentration of 3–4 × 10^6^ cells per ml. The extracellular solution contained [in mM]: 145 NaCl, 4 KCl, 10 CaCl_2_, 10 HEPES, 20 TEA, 1 4-AP and 0.1 Cd^2+^, pH 7.4. The intracellular solution contained [in mM]: 120 CsF, 20 CsCl, 5 NaCl, 10 HEPES and 10 EGTA, pH 7.2. Recordings were obtained at room temperature. Between test pulses, the cells were held in whole-cell clamp at a holding potential of − 80 mV. For TTX and lidocaine experiments, cells were hyperpolarized to − 120 mV for 200 ms, before they were depolarized with ten pulses to 0 mV for 10 ms with an interval of 100 ms between each pulse. For the recordings with ICA-121431, a selective voltage-gated sodium channel (Na_V_) antagonist, a pulse protocol was used which inactivated 50% of the Na_V_ channels to achieve a selective inhibition of the Na_V_ channels (McCormack et al. 2013). Therefore, a pre-pulse to − 55 mV for 500 ms was applied corresponding to the half-inactivation voltage of the cells’ Na_V_ channels, followed by a test pulse to 0 mV for 10 ms. The holding potential between the pulses was − 120 mV. For the biophysical characterization of the activation properties of Na_V_, the cells were kept at a holding potential of − 90 mV. Prior to the test pulse, the cells were hyperpolarized to − 120 mV for 200 ms. The test pulse had a duration of 100 ms and was increased from − 70 mV to + 40 mV in + 10 mV steps. To examine the characteristics of the steady-state inactivation of Na_V_, the cells were stimulated with a test pulse to 0 mV for 10 ms after a pre-pulse with voltage steps from − 110 mV to − 10 mV in + 10 mV steps, which lasted for 500 ms. The recovery from inactivation of Na_V_ was investigated as follows: cells were held at a potential of − 120 mV followed by a first reference test pulse to 0 mV for 200 ms, which was followed by a second test pulse to 0 mV for 20 ms. The time between the two test pulses was increased from 1 ms with a factor of 2.5 in nine steps.

### Manual patch clamp recordings

Manual patch clamp was executed with an EPC 10 USB patch clamp amplifier and PatchMaster Software (version 2 × 90.5; HEKA Elektronik, Lambrecht, Germany). Extracellular solution contained [mM]: 140 NaCl, 4 KCl, 1 MgCl_2_, 1.8 CaCl_2_, 10 HEPES and 10 D-glucose, pH 7.4. Intracellular solution contained [mM]: 107 K-gluconate, 10 KCl, 1 MgCl_2_, 10 HEPES, 5 EGTA, 4 Na_2_ATP and 0.2 NaGTP, pH 7.20. Recordings were obtained at room temperature. The cells were kept at a holding potential of − 70 mV. In voltage clamp mode Na_V_ and voltage-gated potassium (K_V_) channels were activated for 300 ms by voltage pulses ranging from − 70 to + 70 mV in + 10 mV steps after a hyperpolarizing step to − 120 mV for 200 ms. To investigate the firing behavior, cells were stimulated in current clamp mode by hyper- and depolarizing current pulses of 300 ms duration. The pulse protocol was executed at 0.2 Hz. For agonist tests in current clamp and voltage clamp mode, cells were kept at a holding potential of − 70 mV and the compounds were applied for 5 s.

### Data analysis

After offset correction using the FDSS software (version 3.2), the Ca^2+^-imaging data were exported and further analyzed with scripts written in R (version 3.6.3) (R Core Team 2020). The concentration–response curves were fitted using a log-logistic model described by Ritz et al. (2015), utilizing the R package *drc* with its function *drm()* and *LL2.2()* with the following equation: *f*(*x*) = *d*/[1 + exp(*b*(log(*x*) − *ẽ*))] (Ritz et al. 2015). The logarithm of the half-maximal effective concentration (logEC_50_) between 0 and the upper limit (*d*), which was set to 1, is represented by *ẽ*, *x* denotes the concentration and *b* stands for the slope parameter (Ritz et al. 2015). In cases with normalizations to responses induced by another compound, the function *LL2.3()* was used with a variable upper limit (*d*; Ritz et al. 2015). The same equation was used to determine the half-maximal inhibitory concentration (logIC_50_). Then the logEC_50_ and logIC_50_ values were converted into the pIC_50_ and pEC_50_ values, which are the negative logarithms to base 10.

Automated patch clamp data were pre-processed using the QPatch Assay Software (version 5.0) (Sophion Bioscience, DK) for offset correction and to detect the peak currents of the Na_V_ channels. For further analysis, scripts written in R were utilized. For the calculation of the voltage-dependent conductance (*G*/*G*_max_), the peak currents (*I*) were divided by the difference of the stimulation voltage (*V*) and the reversal potential (*V*_rev_) using the equation: *G* = *I*/(*V* *−* *V*_rev_) and then normalized to the maximal conductance (*G*_max_; Cheng et al. 2011; Zhang et al. 2013; Wang et al. 2015). For steady-state inactivation (*I*/*I*_max_), the peak currents were normalized to the maximal peak current. The analyzed data of the voltage-dependent conductance (*G*/*G*_max_) and the steady-state inactivation (*I*/*I*_max_) of Na_V_ were fitted with the Boltzmann equation: *G*/*G*_max_ and *I*/*I*_max_ = 1/(1 + exp[(*V*_50_ − *V*)/*k*]), which was used to estimate the half-maximal voltage (*V*_50_) and the slope factor (*k*; Cheng et al. 2011; Zhang et al. 2013; Wang et al. 2015). For the analysis of the recovery from inactivation, the peak current of the second test pulse (*I*_peak2_) of each time interval was normalized to the peak current of the first reference test pulse (*I*_peak1_) of the corresponding time interval. Data were fitted with a bi-exponential function resulting in a fast and a slow time constant using the equation: *I*_peak2_/*I*_peak1_ = *A*_1_ [1 − exp(− *t*/*τ*_1_)] + *A*_2_ [1 − exp(−*t*/*τ*_2_)] (Zhang et al. 2013). The parameter *t* stands for the time interval, *A*_1_ and *A*_2_ are the amplitudes and *τ*_1_ and *τ*_2_ represent the recovery time constants.

The raw data of the manual patch clamp recordings were analyzed in scripts written in R. For leak subtraction, the P/4 algorithms of PatchMaster and QPatch Software were used in the voltage clamp recordings for manual and automated patch clamp experiments, respectively.

The following r packages were utilized for data handling: cowplot (Wilke 2019), dplyr (Wickham et al. 2020), drc (Ritz et al. 2015), ephys2 (Danker 2018), ggplot2 (Wickham 2016), htmlwidgets (Vaidyanathan et al. 2019), lemon (Edwards 2019), magick (Ooms 2020), magrittr (Bache and Wickham 2014), matrixStats (Bengtsson 2020), miniUI (Cheng 2018), modelr (Wickham 2020), multcomp (Hothorn et al. 2008), plotrix (Lemon 2006), proto (Grothendieck et al. 2016), shiny (Chang et al. 2020), shinyjs (Attali 2020), shinyTree (Trestle Technology, LLC 2017), tidyverse (Wickham et al. 2019).

The raw count tables of gene expression profiling with TempO-Seq assay and traditional whole genome RNA sequencing (RNAseq) were analyzed with the R package DESeq2 (v1.24.0) (Love et al. 2014). RNAseq counts were normalized to the library size and the transcript length (Transcripts per kilobase million (TPM)) (Wagner et al. 2012). TempoSeq counts were normalized to total counts per sample [counts per million (CPM)]. Gene lengths were retrieved from the hg18 reference genome (NCBI Build 36.1) with the R package Goseq (v1.40.0) (Young et al. 2010). TPM/CPM were averaged over the five biological replicates.

### Data handling and statistics

Unless mentioned differently, values are presented as mean ± SEM. If not indicated otherwise, experiments were performed with at least three technical replicates per condition. Detailed data on pEC_50_, pIC_50_ and n numbers are found in supplementary tables. Statistical significance was defined as *P* < 0.05 and was determined by one-way ANOVA with Dunnett’s post hoc test as indicated.

## Results and discussion

### Implementation of high-throughput Ca^2+^-signaling and application to purinergic receptor profiling

A neurofunctional test should ideally be able to assess neuronal activity changes related to various effector systems that directly affect the membrane potential, and thus the neuronal firing properties. Some of the most important toxicant targets are voltage-dependent ion channels, ligand-gated ion channels and electrogenic transporters. The classical neurotoxicological approach uses electrophysiological techniques. These are resource intensive, can only be performed with equipment not readily available in most cell culture laboratories, and require specialist knowledge to perform and interpret the experiments. We explored here whether an imaging-based approach could be used alternatively to capture various neuronal responses in a high-throughput fashion. Notably, the term “high throughput” is here not meant to imply assessment of very large compound collections. We rather see it as important to allow good in vitro toxicology practice, i.e., recording of full concentration–response curves with sufficient replicates (i.e., at least 20–30 data points per compound). Such data sets are still hard (or very expensive) to obtain with patch clamp approaches or microelectrode arrays (MEA). As readout, we used here the rapid changes (peaks) of [Ca^2+^]_i_, because this can be measured easily by imaging devices (in cells loaded with fluorescent indicators), and as this end point is sensitive to changes in the membrane potential that lead to action potentials in neurons.

Initially, we explored purinergic (P2) receptors as example for ligand-gated ion channels. They play an important role in neurons of the peripheral and central nervous system. The P2X family, the focus of our experiments, is present in different types of neurons, for example in dopaminergic neurons. They take various roles in pathological conditions like Parkinson’s disease and in pain mediation (Burnstock and Kennedy 1985; Khakh et al. 2001; Abbracchio et al. 2006; Amadio et al. 2007; Syed and Kennedy 2012; Puchałowicz et al. 2014; Tóth et al. 2019).

**Fig. 1:**
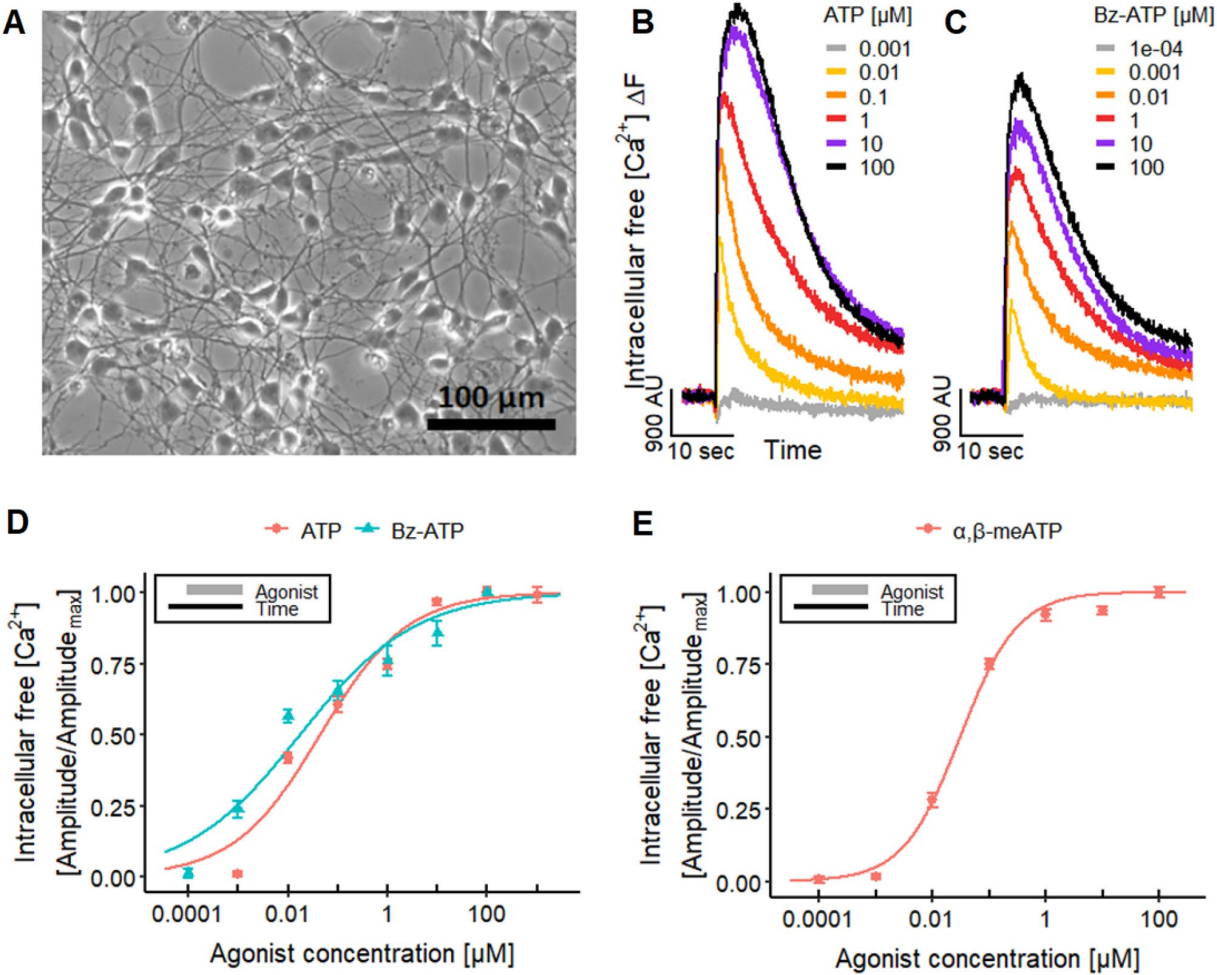
Effects of different P2 receptor agonists. **a** LUHMES cells were differentiated for 7 days on a pre-coated glass coverslip before a phase contrast image was taken. **b**, **c** Ca^2+^-imaging traces displaying the responses of the LUHMES neurons to the application of **b** ATP and **c** BzATP. **d** The concentration–response curves yield pEC_50_s of 7.35 ± 0.08 for ATP and 7.76 ± 0.11 for BzATP in Ca^2+^-imaging. **e** Concentration-dependent effect of α,β-meATP in Ca^2+^-imaging, resulting in a pEC_50_ value of 7.52 ± 0.03. The concentration–response curve depicts the mean of five differentiations of the LUHMES neurons. See Fig. S3D for more details on the curves of the single differentiations. Note the treatment schemes (upper left corner), illustrating the experimental design. Detailed data on n numbers are found in table S4

To obtain reference data, we performed manual patch clamp recordings to investigate the presence of P2X receptors on a single-cell level. LUHMES cells were plated on glass coverslips, where they adhered and extended long neurites (Fig. 1a). The application of ATP triggered action potential firing (Fig. S1A), associated with fast-inactivating inward currents (Fig. S1B). The shape of the current curves indicates the presence of P2X1 or P2X3 receptors (Bianchi et al. 1999; Koshimizu et al. 2000; North 2002; Li et al. 2013). More detailed follow-up experiments suggest that also some receptors with slow inactivation kinetics (e.g., P2X4 or P2X7) (Bianchi et al. 1999; Koshimizu et al. 2000; North 2002; Li et al. 2013) may be present (Fig. S1C, D). Gene expression studies on the LUHMES cells confirmed the presence of mRNA of several P2X receptors (Fig. S2), with P2X3 appearing to be dominant.

On this basis, we tried to assess the responses induced by the activation of P2X receptors using Ca^2+^-imaging. LUHMES loaded with a [Ca^2+^]_i_ indicator dye were exposed to the endogenous P2X receptor agonist ATP (Fig. 1b) and the synthetic ATP analog BzATP (Fig. 1c) (Bianchi et al. 1999; Khakh and North 2012). A strong signal peak was recorded, and the fast decrease of the fluorescence signal (half-life of ~ 8 s at 100 µM ATP) found here is typical for P2X1 and P2X3 receptors as reported previously in GT1-7 cells (Koshimizu et al. 2000; He et al. 2003) (Fig. 1b). We obtained pEC_50_ values from seven-point concentration–response curves (*n* = 42 data points per compound) (Fig. 1d), and these were well in line with published data for human P2X1 and P2X3 receptors.

As our results suggest a strong contribution of P2X1 and P2X3 to the responses induced by ATP, we used this as an example for a mechanistic follow-up of an observed effect. A specific agonist of these receptors (i.e., α,β-meATP) was employed to confirm their involvement (Burnstock and Kennedy 1985; Abbracchio and Burnstock 1994; Bianchi et al. 1999; Gever et al. 2006; Khakh and North 2012). We first obtained reference data by manual patch clamp. The electrophysiological responses (Fig. S3A, B) suggested the activation of P2X1 and P2X3 receptor subtypes (Bianchi et al. 1999; Koshimizu et al. 2000; Burgard et al. 2000; North 2002; Li et al. 2013). The selective agonist α,β-meATP also led to a cellular response in Ca^2+^-imaging experiments (Fig. S3C). The recordings were performed for five differentiations at seven concentrations to illustrate the reproducibility of the test system and end point (Fig. S3D). The pEC_50_ value of 7.5 (Fig. 1e) and the fast inactivation of the fluorescence signal (Fig. S3C) confirmed a functional expression of P2X1 and/or P2X3 receptors (Bianchi et al. 1999; Gever et al. 2006).

For a biological calibration of the α,β-meATP signal, we compared it to the signal triggered by an increase in the buffer K^+^ concentration (maximal depolarizing stimulus). The response evoked by the purinergic agonist reached up to 28% of the K^+^-induced signal intensity. This is well in line with P2X receptor activation being a physiological response that does not reach the level of complete and irreversible cell depolarization (Fig. S3E, F).

**Fig. 2:**
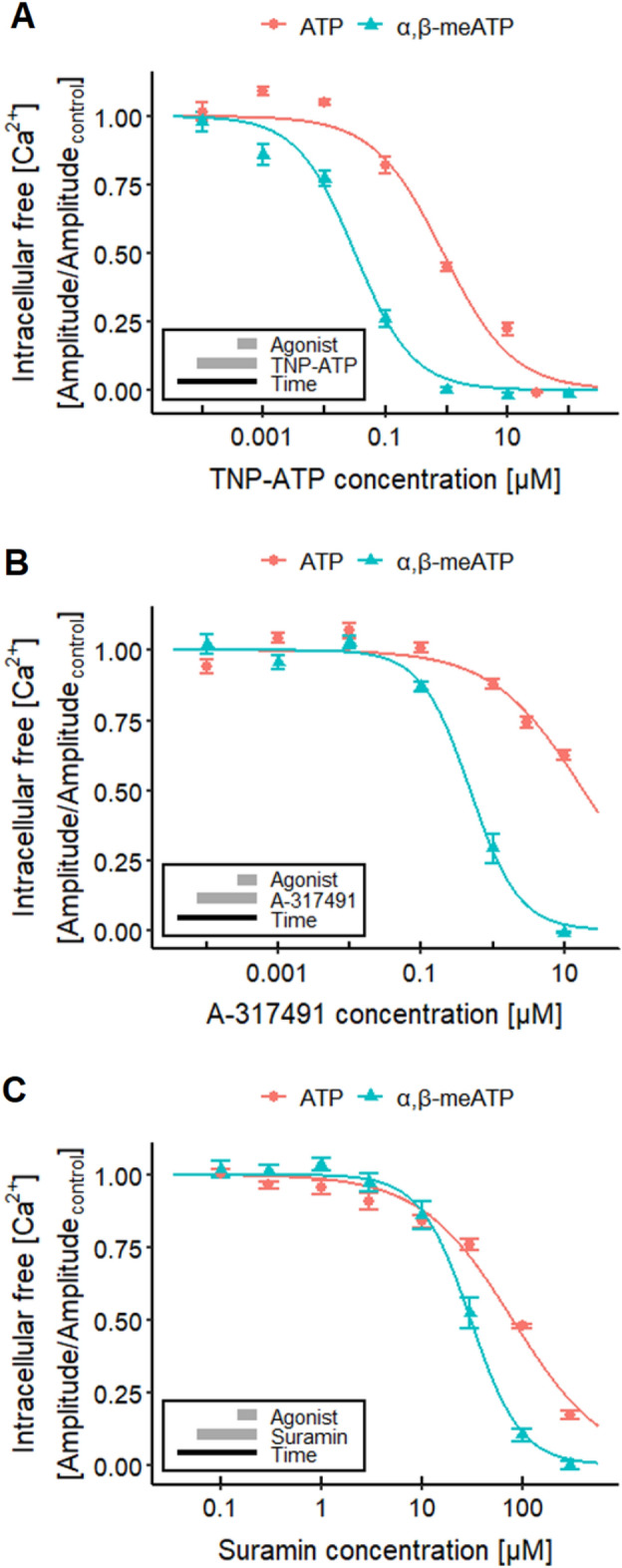
Characterization of P2X receptors. Ca^2+^-imaging experiments researching the effects of different P2X receptor antagonists on the response of LUHMES cells triggered by 1 µM ATP (~ EC_75_, Fig. 1d) and 0.1 µM α,β-meATP (~ EC_75_, Fig. 1e). **a** The responses were blocked concentration-dependently by TNP-ATP with pIC_50_s of 6.05 ± 0.06 for ATP and of 7.50 ± 0.05 for α,β-meATP. The values are significantly different. **b** A-317491 blocked the responses of ATP up to 40% and yielded a pIC_50_ for α,β-meATP of 6.31 ± 0.05. **c** An inhibitory effect of suramin could be detected for ATP and α,β-meATP, which resulted in pIC_50_s of 4.09 ± 0.03 and 4.51 ± 0.03, respectively. Note the treatment schemes (lower left corner), illustrating the experimental design. Detailed data on n numbers are found in table S4

As a next step to characterize the suitability of the test system, we studied interference with Ca^2+^-signaling. The antagonistic effect of TNP-ATP (competitive antagonist) on the response to ATP and α,β-meATP was compared in Ca^2+^-imaging experiments (Fig. 2a). At high concentrations, we observed a complete block of signaling, and the concentration–response features of the antagonist were agonist dependent, as expected for a competitive inhibitor. The significant difference of pIC_50_ values (6.1 and 7.5) suggests that ATP stimulates a broad panel of P2X receptors (for some of which TNP-ATP has a relatively low affinity) (Virginio et al. 1998; Gever et al. 2006). To further explore how well such differential antagonist effects can be described and quantified, we used a selective antagonist of P2X3 receptors, A-317491 (Virginio et al. 1998; Gever et al. 2006). This compound potently and completely blocked the response to α,β-meATP, while it showed only a weak partial effect on ATP signals (Fig. 2b).

It needs to be noted here that ATP, but not α,β-meATP, can also activate P2Y receptors (Abbracchio et al. 2006; von Kügelgen and Harden 2011), for example P2Y1 (Palmer et al. 1998; Waldo and Harden 2004) and P2Y11 receptors (Communi et al. 1999; Qi et al. 2001; White et al. 2003), which are also expressed in LUHMES neurons and which may theoretically act as response modifiers (Fig. S2).

Finally, we used the antiprotozoal agent suramin to show applicability of the LUHMES system to characterize xenobiotics potentially interfering with P2X receptors. One known side effect of suramin, a drug used for the treatment of sleeping sickness (Wéry 1994; Kennedy 2013), is the inhibition of P2X receptors (Garcia-Guzman et al. 1997; Gever et al. 2006; Coddou et al. 2011; Khakh and North 2012). The experiments yielded pIC_50_s of 4.1 for ATP and 4.5 for the P2X3 ligand α,β-meATP (Fig. 2c). The data obtained with α,β-meATP agree with a previously reported pIC_50_ of approximately 4.8 for human P2X3 receptors (Garcia-Guzman et al. 1997).

In summary, these initial experimental system evaluations confirmed that measurement of [Ca^2+^]_i_ in LUHMES can capture (some) electrophysiological responses related to drug effects and toxicity. The data available at this stage indicate that the potency quantifications for agonists and antagonists are precise (little variation between cell preparations) and exact (very similar to reference systems).

### Characterization of Na_V_ channel toxicants

Next, we moved to the detection of potential channel modulators and checked whether agents affecting voltage-dependent sodium channels (Na_V_ channels) could be characterized by Ca^2+^-imaging. Na_V_ channels are essential for the onset of action potentials and thus are of critical importance for the neuronal electrical activity. An alteration of their function can lead to severe functional neurotoxicity, even with lethal consequences on the level of the organism (Lehane and Lewis 2000; Gaillard and Pepin 2001; Nicholson and Lewis 2006; Llewellyn 2009; Wiese et al. 2010; Vilariño et al. 2018; Anwar et al. 2018). Na_V_ channels couple to [Ca^2+^]_i_ indirectly by triggering cell depolarization, which in turn leads to the opening of voltage-dependent calcium channels and thus an influx of Ca^2+^ into neurons (Vetter et al. 2012; Mohammed et al. 2017).

**Fig. 3:**
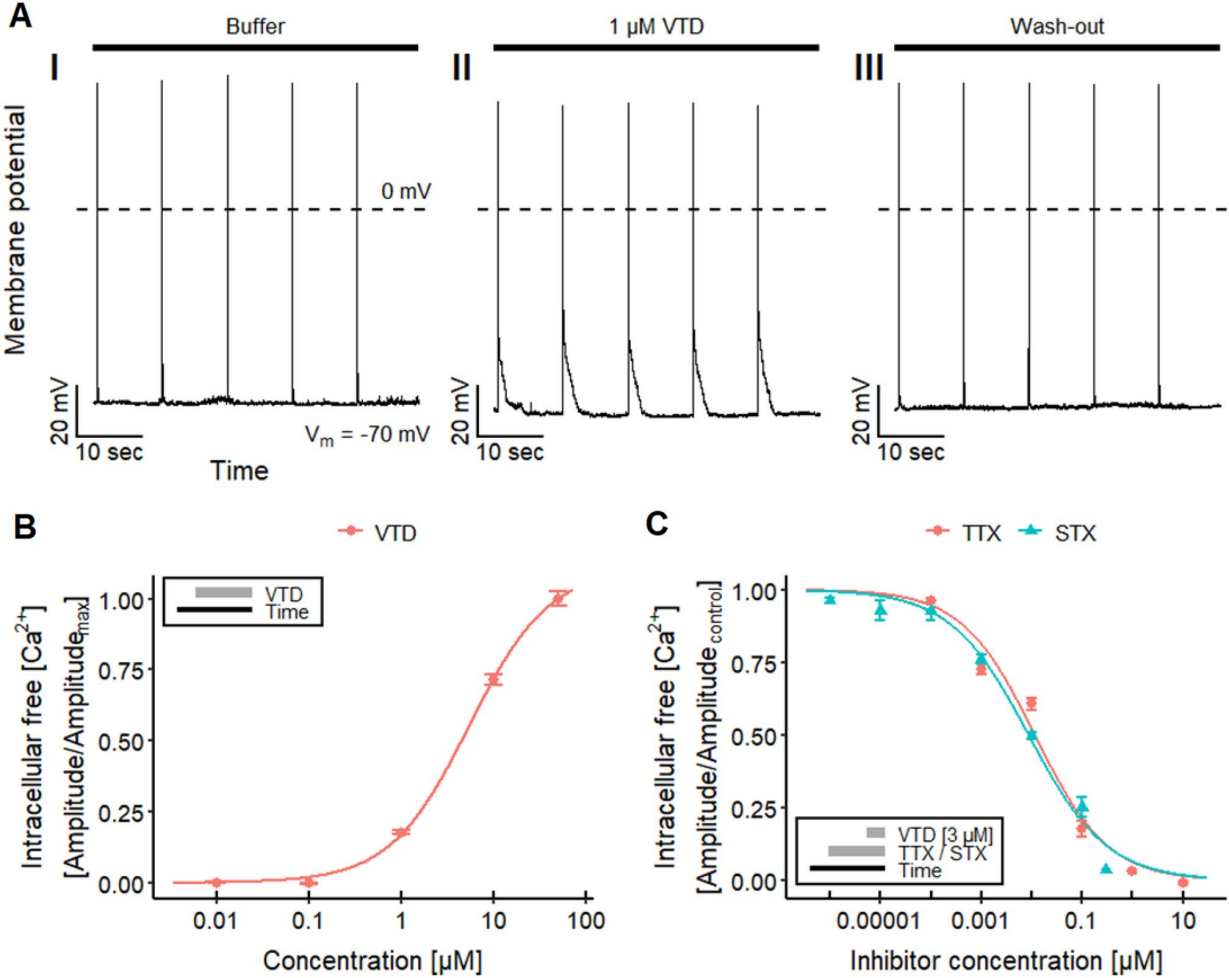
Na_V_ channel toxicity—effect of veratridine (VTD). **a** Traces of a manual patch clamp experiments with LUHMES neurons showing five action potentials, triggered by 10 ms depolarizing current pulses with 0.1 Hz, in untreated control (I), in the presence of 1 µM veratridine (VTD, II) and after wash-out (III, *n* = 4). VTD reversibly increased the action potential duration. **b** Ca^2+^-imaging experiments demonstrating the concentration-dependent effects of VTD on LUHMES neurons, which yielded a pEC_50_ value of 5.38 ± 0.03. **c** Concentration-dependent inhibitory effect of TTX and STX on the response triggered by 3 µM VTD, resulting in pIC_50_ values of 7.93 ± 0.06 for TTX and 8.06 ± 0.06 for STX. Note the treatment schemes, illustrating the experimental designs. Detailed data on n numbers are found in table S4

To check practical applicability, we studied the Ca^2+^-response of well-known Na_V_ channel toxicants. First, veratridine (VTD), a plant alkaloid (Ulbricht 1998; Wang and Wang 2003) known to delay Na_V_ channel inactivation (Catterall 1992; Power et al. 2012; Tsukamoto et al. 2017; Zhang et al. 2018), was used. For background information, patch clamp data were obtained and VTD reversibly increased the action potential duration (Fig. 3a). Imaging experiments then showed a concentration-dependent rise of [Ca^2+^]_i_ that resulted in a pEC_50_ value of 5.4 for VTD (Fig. 3b). These data are in good agreement with published data on human SH-SY5Y cells (Vetter et al. 2012). Also here, we moved on to also study antagonism: VTD-induced responses were blocked by the Na_V_ channel antagonist tetrodotoxin (TTX, Fig. S4A) with a pIC_50_ of 7.9 (Fig. 3c). The inhibition of VTD-mediated effects by TTX is consistent with previously reported data from patch clamp recordings on rat hippocampal neurons (Alkadhi and Tian 1996), Ca^2+^-imaging experiments on mouse DRG neurons (Mohammed et al. 2017) and human SH-SY5Y cells (Vetter et al. 2012).

As additional proof-of-concept test compound, we used saxitoxin (STX), an alkaloid that is produced by certain genera of cyanobacteria and marine dinoflagellates (Deeds et al. 2008; Westrick et al. 2010; Wiese et al. 2010; He et al. 2016). It can contaminate water supplies and accumulate in the marine food chain, and thus cause paralytic shellfish poisoning in humans (Deeds et al. 2008; Wiese et al. 2010; Durán-Riveroll and Cembella 2017). Like TTX, STX is known for its inhibitory effect on the Na_V_ channels (Noda et al. 1989; Terlau et al. 1991; Llewellyn 2009; Mattei and Legros 2014; Durán-Riveroll and Cembella 2017). We tested the effect of STX on the response evoked by 3 µM VTD to verify the capability of our assay to detect Na_V_ channel-modulating biotoxins (Fig. S4B). The pIC_50_ value of 8.1 (Fig. 3c) found here is comparable to data previously described for recordings with rat Na_V_1.2 expressed in *Xenopus laevis* oocytes (Noda et al. 1989). These results demonstrate the capability of setting up a LUHMES cell-based assay for the detection of biotoxins, which affect Na_V_ channel activity, and the quantification of their effects, using Ca^2+^-imaging.

To further evaluate the usability of LUHMES cells as relevant functional neurotoxicity model, we examined the effect of pacific ciguatoxin-2 (pCTX-2) on the LUHMES cells via Ca^2+^-imaging (Fig. S4C–E). The ciguatoxins are marine biotoxins synthetized by dinoflagellates (Lehane and Lewis 2000; Nicholson and Lewis 2006; Litaker et al. 2010, 2017; Vilariño et al. 2018). These polycyclic ethers accumulate in the marine food chain and can lead to intoxications in humans, called ciguatera, after consumption of CTX-contaminated fish (Lehane and Lewis 2000; Nicholson and Lewis 2006; Dickey and Plakas 2010; Skinner et al. 2011; Vilariño et al. 2018). The CTXs comprise several analogs, with an acute toxicity (mice) of pCTX-2 of 2.3 µg/kg (Lewis et al. 1991; Lehane and Lewis 2000; Nicholson and Lewis 2006). We found in LUHMES neurons that 15 nM pCTX-2 increased [Ca^2+^]_i_. This response was followed by long-lasting oscillations of the Ca^2+^-imaging signal. The initial increase of the baseline Ca^2+^ level is likely explained by Na^+^ influx, followed by entry of Ca^2+^ into the cytosol through Ca_V_ channels (Molgó et al. 1993). The oscillation of the Ca^2+^-imaging signal is noteworthy as our data are not single-cell recordings, but measures of [Ca^2+^]_i_ in all cells of the whole neuronal network. It is thus a first indication of synchronized activity of the LUHMES neuron population (see more details below). Notably, it has been shown earlier that CTX-1 can induce oscillations of the membrane potential and repetitive firing of action potentials in other cells (Bidard et al. 1984; Hamblin et al. 1995; Hogg et al. 1998, 2002; Birinyi-Strachan et al. 2005).

Finally, we also explored the effects of a microcystin to test for the specificity of the LUHMES test system. Microcystins are cyclic peptides produced by a number of cyanobacteria genera (Sivonen and Jones 1999; He et al. 2016) found for example in contaminated water and fish (Campos and Vasconcelos 2010). They inhibit protein phosphatases, like PP1 and PP2A (MacKintosh et al. 1990; Campos and Vasconcelos 2010), but are not known to affect ion channels. We therefore anticipated that microcystin-LF (a potent hepatotoxicant) would not alter the response of the LUHMES cells. We tested the effect of a high concentration of 2 µM microcystin-LF, alone and on the response to 3 µM VTD (Fig. S4F). The toxin did not show any effects in our Ca^2+^-imaging assay, as expected. This result illustrates the capability of our assay to distinguish between biotoxins directly affecting the electrical activity of neurons and toxins that exhibit a different, cytotoxic mode of action.

In summary, our findings show the capability of the LUHMES test system to detect biotoxins that affect Na_V_ channel activity in an agonistic or antagonistic way. Taken together, the experiments on P2X receptors and Na_V_ channels demonstrated that whole-culture, high throughput Ca^2+^-imaging is a suitable assay end point to broadly cover various types of functional neurotoxicants.

### Electrophysiological characterization and pharmacological modulation of Na_V_

Before moving on with exploring further types of potential toxicant targets, we considered it important to provide a basic electrophysiological characterization of the LUHMES cultures. As the cells are derived from a cell line, it was important to confirm that all cells in culture consistently show genuine neuronal electrical properties (as assessed here by the generation of action potentials). A comprehensive investigation of firing behavior (*n* = 274 cells) showed that 45% of cells displayed phasic (Fig. S5A) and 51% a tonic firing pattern (Fig. S5B). Depolarization failed to induce action potentials in only 4% of the cells. Such a co-occurrence of phasic and tonic action potential firing behavior has also been described for primary dopaminergic neurons (Grace and Bunney 1984a, b). These findings support our earlier data on few selected cells (Scholz et al. 2011), and provide clear proof for functional expression of voltage-gated ion channels throughout the whole population.

**Fig. 4:**
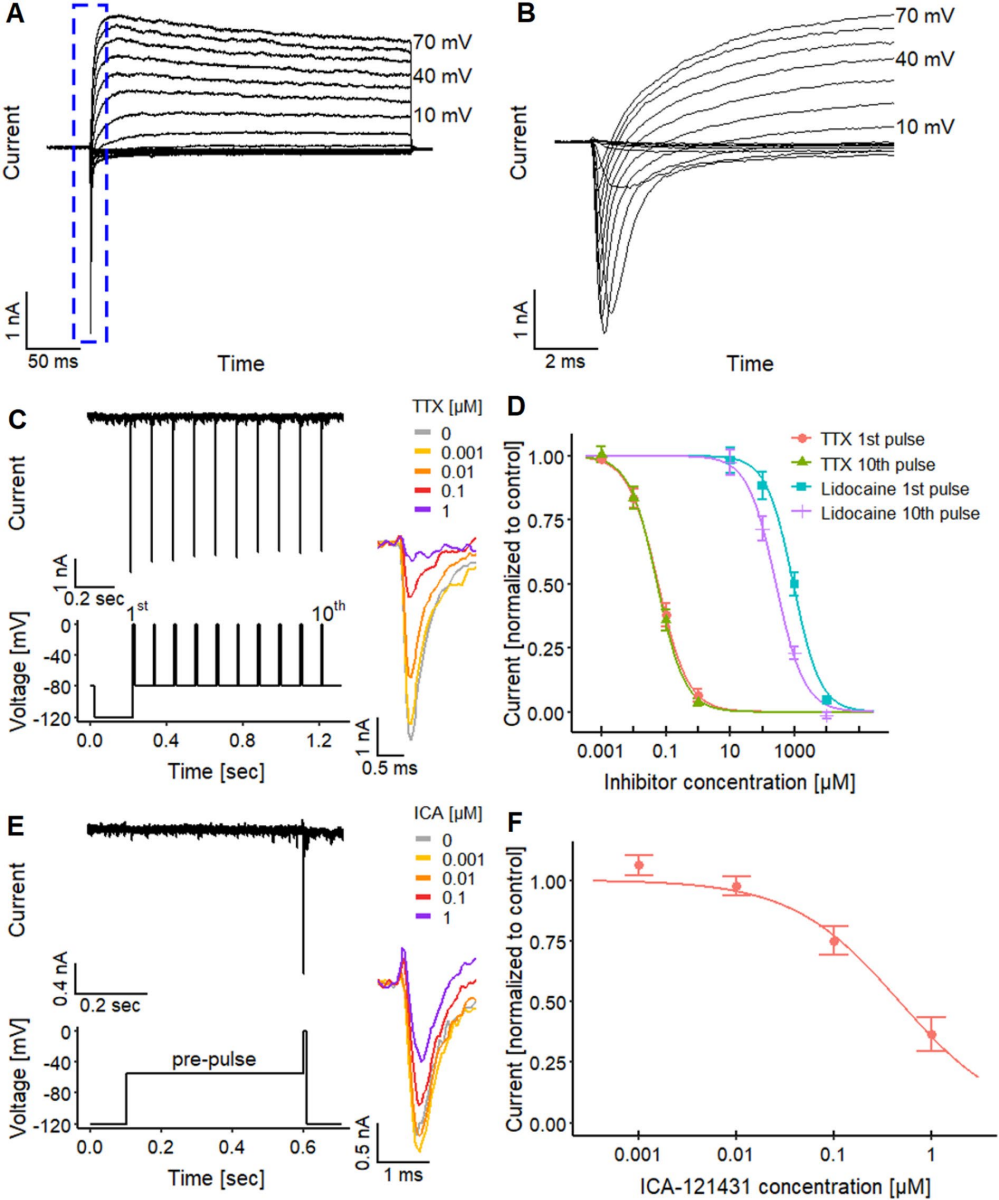
Electrophysiological characteristics of Na_V_ channel. **a**, **b** Manual patch clamp recordings of **a** activation of voltage-gated inward and outward currents stimulated by different voltage steps with **b** a magnification of the inward current (note: different time axis). **c**–**f** Automated patch clamp recordings for a pharmacological characterization of voltage-gated Na^+^ (Na_V_) channels, using **c**, **d** TTX and lidocaine to investigate the use-dependent and acute inhibitory effects and **e**, **f** ICA-121431 to narrow Na_V_ channel subtypes down. **e** Exemplary trace of the inward current triggered by the pulse protocol with ten closely spaced test pulses. Traces on the right depict the concentration-dependent effect of TTX on the 1st pulse. **d** Concentration–response curves yielded pIC_50_ values of 7.23 ± 0.05 (1st pulse) and 7.26 ± 0.05 (10th pulse). Lidocaine had pIC_50_ values of 3.03 ± 0.07 (1st pulse) and 3.57 ± 0.06 (10th pulse). The pIC_50_ values for lidocaine are significantly different, unlike the pIC_50_ values of TTX. **e** To ensure a selective effect of ICA-121431, measurements need to be performed under conditions where 50% of the Na_V_ channels are inactivated (McCormack et al. 2013). This was achieved by the application of a prepulse to − 55 mV before the stimulus, the determined V_50_ value for steady-state inactivation in these cells (Fig. S5F). Traces on the right depict the concentration-dependent effect of ICA-121431. **f** Effect of ICA-121431 with a pIC_50_ value of 6.33 ± 0.10. Detailed data on *n* numbers are found in table S4

When cells were exposed to depolarizing voltage pulses (voltage clamp recordings), rapid transient inward currents were observed when the membrane potential was raised to levels higher than − 40 mV. This was followed by long-lasting outward currents (Fig. 4a, b). The inward currents were blocked by TTX (1 µM). Outward currents were inhibited by a combination of TEA (10 mM intra- and extracellularly) together with the replacement of K^+^ with Cs^+^ in the intracellular solution (data not shown) to prevent current flow through K_V_ channels. Taken together, these data confirmed expression of functional Na_V_ and K_V_ channels, a key feature of excitable neurons. To further provide a solid background description of the test system, we obtained gene expression data for many channel constituents and other genes involved in neuronal signaling. The distinct neuronal features of the cells were also confirmed here (Fig. S2).

To better understand the functional implication of the expression of different Na_V_ channel subtypes, we set out to explore their potential electrophysiological role in LUHMES. Because of the higher throughput than manual patch clamp, we used automated planar patch clamp recordings to obtain detailed biophysical data on the Na_V_ channels. All features were in good agreement with data on Na_V_ expression systems (Fig. S5C–F) (Cummins et al. 2001; McCormack et al. 2013; Oliva et al. 2014; Patel et al. 2016). This coherence also applied to the use dependence of the channels, a feature which can have important toxicological implications (Fig. 4c). Lidocaine was studied here as a well-known example of a use-dependent blocker for Na_V_ channels. It led to significantly different pIC_50_ values of 3.03 (for the 1st peak of a stimulation sequence) vs 3.57 (10th peak of the sequence, Fig. 4d). The higher potency of lidocaine on the tenth Na_V_ current peak indicates a use-dependent mechanism of the inhibition on Na_V_ channels as reported previously (Clarkson et al. 1988; Huang et al. 2006; Leffler et al. 2007). The pIC_50_ values of lidocaine were similar to those obtained for rat hippocampal neurons (~ 3.4) (Kaneda et al. 1989).

As Na_V_ channel subtypes play an important role in toxicology and pharmacology, we used here TTX as tool to distinguish two major classes potentially expressed on LUHMES. The pIC_50_ values found here for TTX were in the low nM range (~ 7.2), indicating the presence of TTX-sensitive Na_V_ channels. To compare these inhibition data to the lidocaine data set, we explored whether TTX effects were use dependent. In contrast to lidocaine, the pIC_50_ for the first and the tenth pulse were identical for TTX (Fig. 4d).

Based on these data, the Na_V_ subtypes of LUHMES may be Na_V_1.1, Na_V_1.2, Na_V_1.3, Na_V_1.4, Na_V_1.6 or Na_V_1.7 (all reported to be TTX-sensitive, Ogata and Ohishi 2002; Lee and Ruben 2008; England and de Groot 2009; Zhang et al. 2013). However, the subtypes Na_V_1.4 and Na_V_1.7 are mainly present in skeletal muscle and in the peripheral nervous system, respectively (Ogata and Ohishi 2002; Lee and Ruben 2008; England and de Groot 2009; Zhang et al. 2013). The remaining subtypes Na_V_1.1, Na_V_1.2, Na_V_1.3 and Na_V_1.6 are strongly expressed in the CNS (Ogata and Ohishi 2002; Lee and Ruben 2008; England and de Groot 2009; Zhang et al. 2013). To further narrow down Na_V_ channel subtypes, the selective Na_V_ channel antagonist ICA-121431 was used. It has been shown to exhibit high potency for Na_V_1.1 and Na_V_1.3 (IC_50_ = 0.023 µM and 0.013 µM, respectively), an intermediate potency for Na_V_1.2 (IC_50_ = 0.240 µM) and low potency for Na_V_1.6 and Na_V_1.7 (IC_50_ = 13 µM and 10 µM), respectively (McCormack et al. 2013). We found a pIC_50_ value of 6.3, (Fig. 4e, f), characteristic for Na_V_1.2 (McCormack et al. 2013). This implies that the mainly active Na_V_ channel subtype in the LUHMES neurons is Na_V_1.2. These findings are consistent with the expression levels of Na_V_ channels (Fig. S2). Data on mRNA levels suggest that Na_V_1.2 and Na_V_1.9 show a time-dependent expression, reaching a maximum on d9. Other channels (e.g., Na_V_1.3 and Na_V_1.8) are also expressed on d9, but do not show the typical developmental up-regulation.

### Assessment of agents interfering with the dopamine transporter (DAT)

As LUHMES are dopaminergic cells, we chose the dopamine transporter (DAT) to exemplify measurements of electrogenic effects of neuronal transporters. This was intended as basis to explore functional neurotoxicity of transporter modulating drugs. To confirm the expression of functional DAT, we examined the uptake of radioactively-labeled DA ([^3^H]DA) into LUHMES cells. There was a fast and specific uptake, as expected (Fig. S6). The uptake of [^3^H]DA was significantly reduced by the DAT inhibitors AMP, GBR12935 (Andersen 1987; Rothman et al. 1993), cocaine (Han and Gu 2006; Schmitt et al. 2013) and nomifensine.

The DAT acts as a symporter of dopamine, two Na^+^ and one Cl^−^ ion, and depends on the electro-chemical gradient of the two ions to transport dopamine into the neurons (Harris and Baldessarini 1973; Kuhar and Zarbin 1978; Krueger 1990; Gu et al. 1994; Sonders et al. 1997; Schenk 2002). This transport can lead to a depolarization of the membrane potential and thereby to an activation of voltage-gated ion channels (Sonders et al. 1997; Sitte et al. 1998; Robertson et al. 2009; Cameron et al. 2015). Cameron et al. (2015), showed recently that the activation of the DAT by dopamine (DA) and amphetamine (AMP) leads to the activation of L-type Ca_V_ channels.

**Fig. 5:**
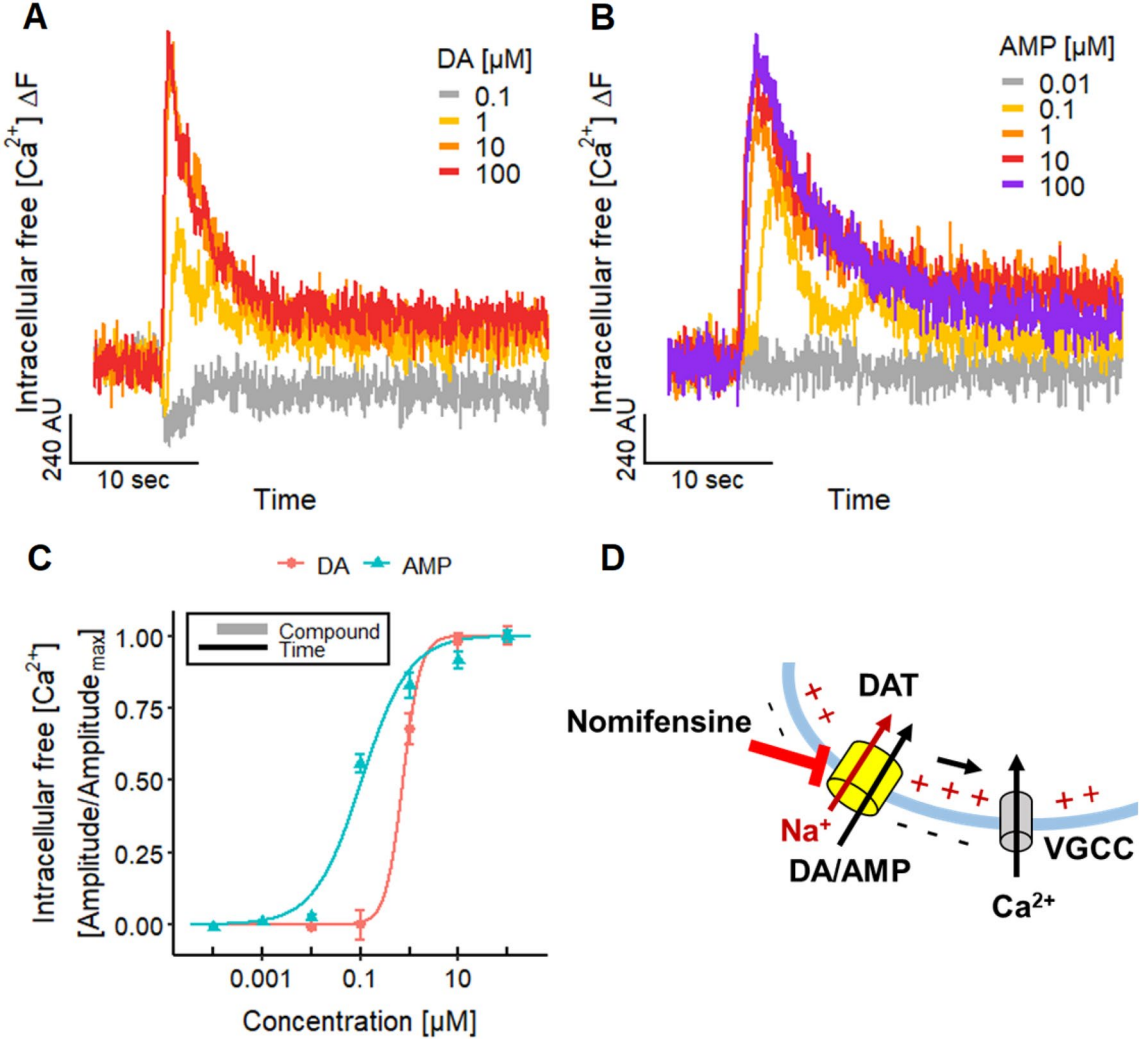
Altered neuronal signaling by DAT substrates. **a**, **b** Traces of Ca^2+^-imaging experiments illustrating the responses of the LUHMES neurons evoked by different concentrations of **a** dopamine (DA) and **b** amphetamine (AMP). **c** Concentration–response curves for DA and AMP with pEC_50_ values of 6.13 ± 0.12 and 6.98 ± 0.06, respectively. Note the treatment scheme (upper left corner), illustrating the experimental design. Detailed data on n numbers are found in table S4. **d** Schematic illustration of the underlying mechanisms of Ca^2+^-imaging signals evoked by DA and AMP. The transport of DA and AMP via the DAT into the cell leads to a net influx of one positive charge (Na^+^) that can active voltage-gated ion channels, like Ca_V_ ion channels. The DAT can be blocked by nomifensine

Measurements of Ca^2+^ showed that this end point can be used to monitor DAT activity in LUHMES cultures: The addition of DA evoked an increase in [Ca^2+^]_i_ (Fig. 5a). Furthermore, the psychostimulant drug amphetamine (AMP), which acts as a substrate of the DAT (Sonders et al. 1997; Sitte et al. 1998; Jones et al. 1998; Fleckenstein et al. 2007; Robertson et al. 2009; Schmitt et al. 2013; Siciliano et al. 2014; Cameron et al. 2015), induced also signals in Ca^2+^-imaging experiments (Fig. 5b) (Cameron et al. 2015). The pEC_50_ of 6.1 and 7.0 for DA and AMP, respectively (Fig. 5c), are in accordance with published findings (Cameron et al. 2015).

**Fig. 6:**
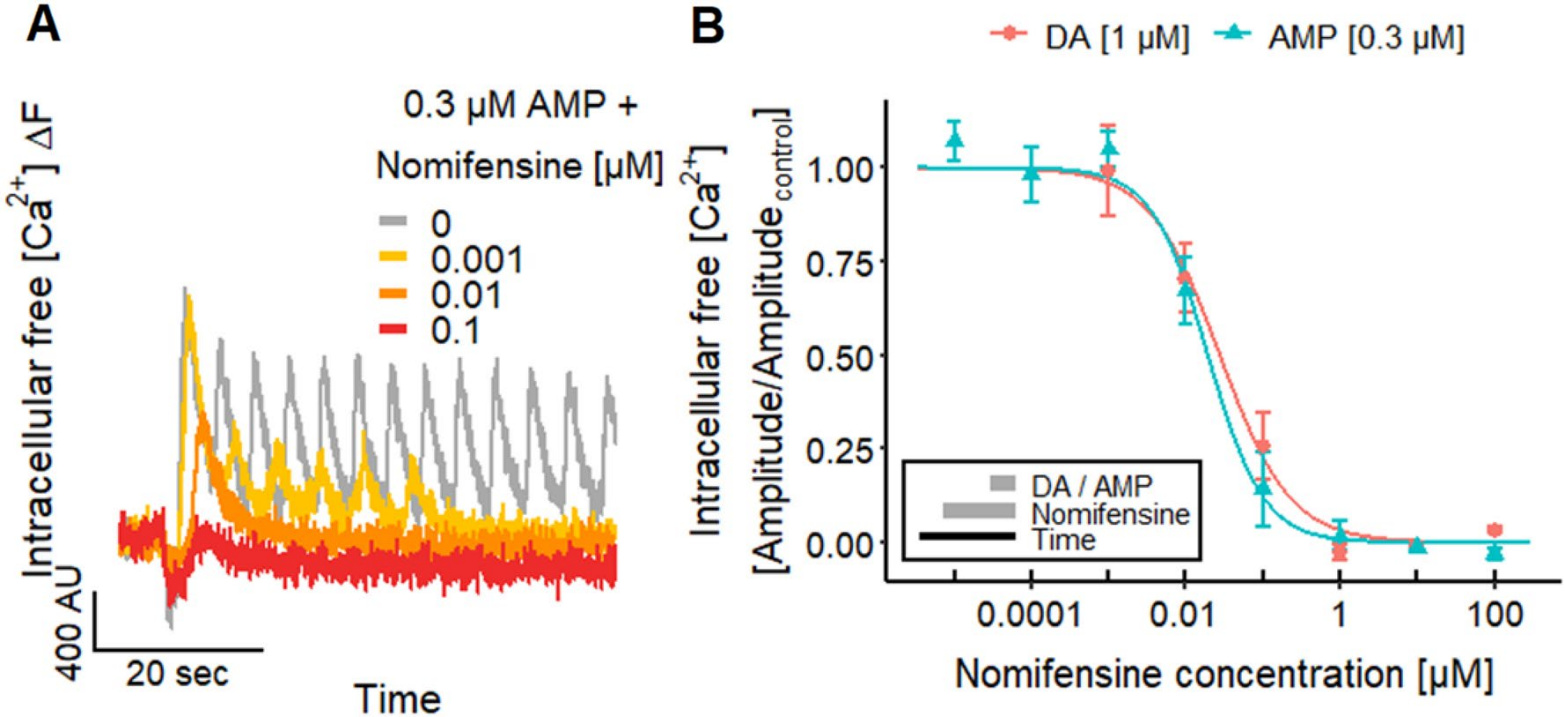
Altered neuronal signaling by blocking the DAT. **a** Ca^2+^-imaging traces of the inhibitory effect of nomifensine on the response of LUHMES neurons to 0.3 µM AMP. **b** The concentration-dependent inhibition by nomifensine resulted in pIC_50_ values of 7.55 ± 0.12 for DA and 7.71 ± 0.10 for AMP. Note the treatment scheme (lower left corner), illustrating the experimental design. Detailed data on n numbers are found in table S4

To make use of the fact that measurements of [Ca^2+^]_i_ can give very exact information on compounds affecting the DAT (Fig. 5d), we explored this approach for characterization of antagonists. The DAT blocker nomifensine (Andersen 1989; Krueger 1990; Sulzer et al. 1995) inhibited the responses evoked by DA and AMP (Fig. 6a) with a pIC_50_ value of 7.6–7.7 (Fig. 6b). This is similar to data on the uptake of radioactively labeled DA in rat synaptosomes (Randrup and Bræstrup 1977).

These results demonstrate the usability of our LUHMES cell-based test system for the assessment of substance-induced changes in DAT activity. The use of high-throughput Ca^2+^-imaging allows the direct detection of DAT-mediated signal changes without requiring special reagents (radioactive labeling to measure [^3^H]DA uptake).

### Ca^2+^-oscillations as indicator of coupled neuronal networks

**Fig. 7:**
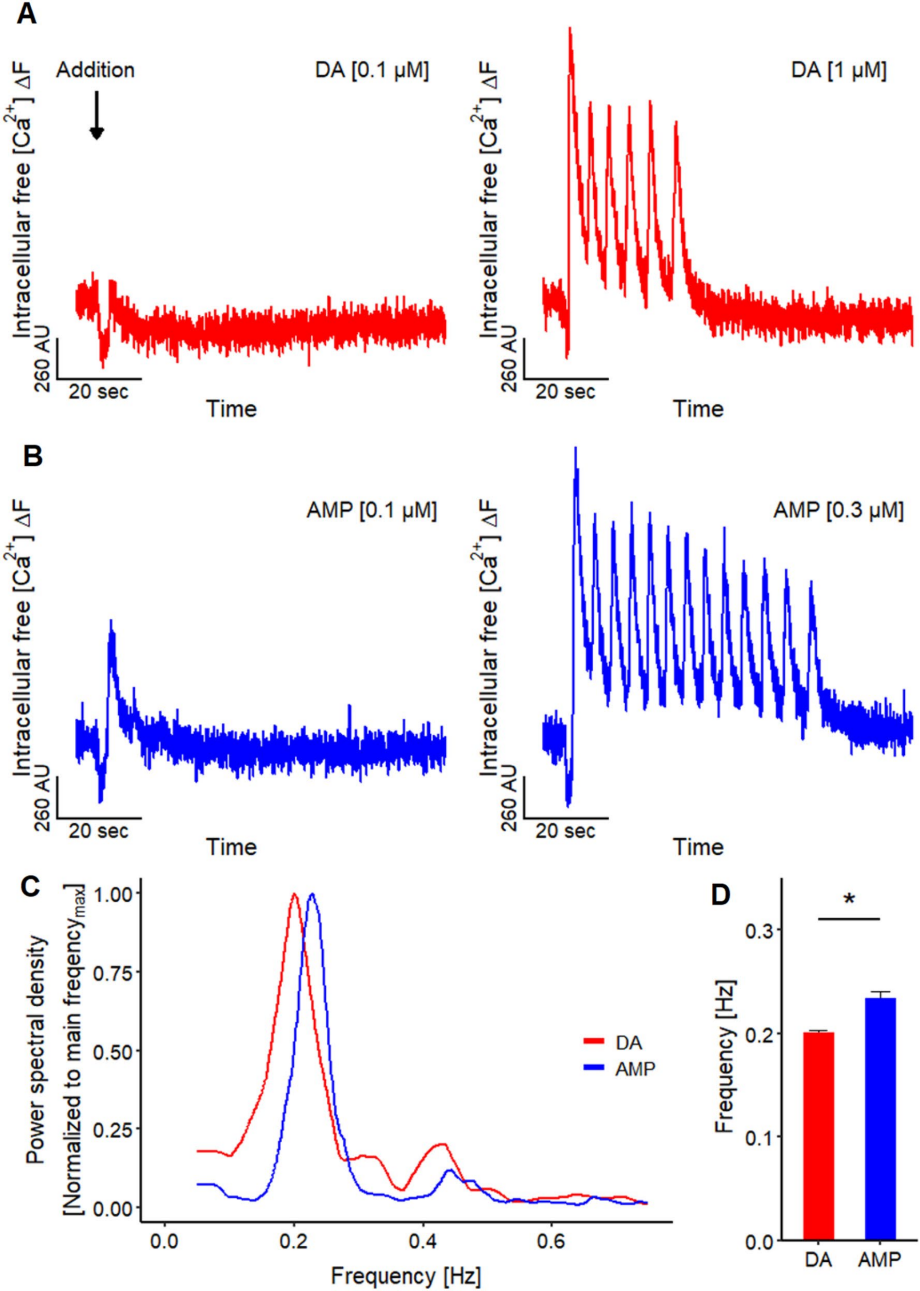
Oscillation of Ca^2+^-imaging signal. **a** Traces of Ca^2+^-imaging signals of the application of 0.1 and 1 µM DA. The latter concentration induced in 61.6% (*n* = 125) of the recordings oscillations of the Ca^2+^-imaging signal. **b** Exemplary traces of the addition of 0.1 and 0.3 µM AMP. Oscillations of the Ca^2+^-imaging signal were induced by the application of 0.3 µM AMP in 94.0% (*n* = 50) of the recordings. **c** Periodogram based on the mean results of FFT analysis of the oscillations induced by 1 µM DA (*n* = 6) and 0.3 µM AMP (*n* = 10) highlighting a main oscillation frequency. **d** Main oscillation frequency for 1 µM DA and 0.3 µM AMP are 0.201 ± 0.002 Hz (*n* = 6) and 0.234 ± 0.007 Hz (*n* = 10), respectively. Statistical significance was determined between DA and AMP (*, significant)

Addition of DA (Fig. 7a) and AMP (Fig. 7b) triggered not only an increase of [Ca^2+^]_i_, but also prominent and long-lasting oscillations of the Ca^2+^-imaging signal. This observation is insofar remarkable, as the [Ca^2+^]_i_-signal was derived from thousands of cells at the same time, and from an area having a diameter of > 2000 µm (for comparison: LUHMES cell bodies are about 20 µm wide). As non-coordinated oscillations of individual cells would cancel out under our measurement conditions (recording of the average signal of all cells), the measurable oscillations indicate that all cells change [Ca^2+^]_i_ in a synchronized way, and that LUHMES cultures must therefore form a functionally coupled network.

We explored how consistent this phenomenon occurred across several wells and cell differentiations: in 62% (*n* = 125) of cases, DA (1 µM) triggered oscillations. When AMP (0.3 µM) was used, oscillations were observed in 48 of 50 experiments. The oscillation frequency was consistent throughout the experiments in a range of 0.2–0.23 Hz (Fig. 7c, d). This means that the entire culture in a well increased the average [Ca^2+^]_i_ about every 5 s in a coordinated way. In summary, these findings suggest that whole culture measurements of [Ca^2+^]_i_ allow assessment of neuronal network properties. An example of a drug triggering network oscillations is given here with AMP.

### Modulation of [Ca^2+^]_i_ oscillations in functionally coupled neuronal cultures

To test the hypothesis that DAT activity is required for the oscillations, we used the DAT blocker nomifensine. The oscillations induced by AMP and DA were indeed blocked by this drug (Fig. 6a). Nomifensine thus exemplifies possible modes of action of drugs that dampen or break network synchronization. However, it was important to test whether oscillations driven by the DAT may also be modified by drugs with other neuronal targets. We therefore asked which types of channels may be involved in ensuring coordinated oscillatory activity in LUHMES cultures, and whether drugs interfering with such channels would affect network oscillations as potential neurofunctional end point.

**Fig. 8:**
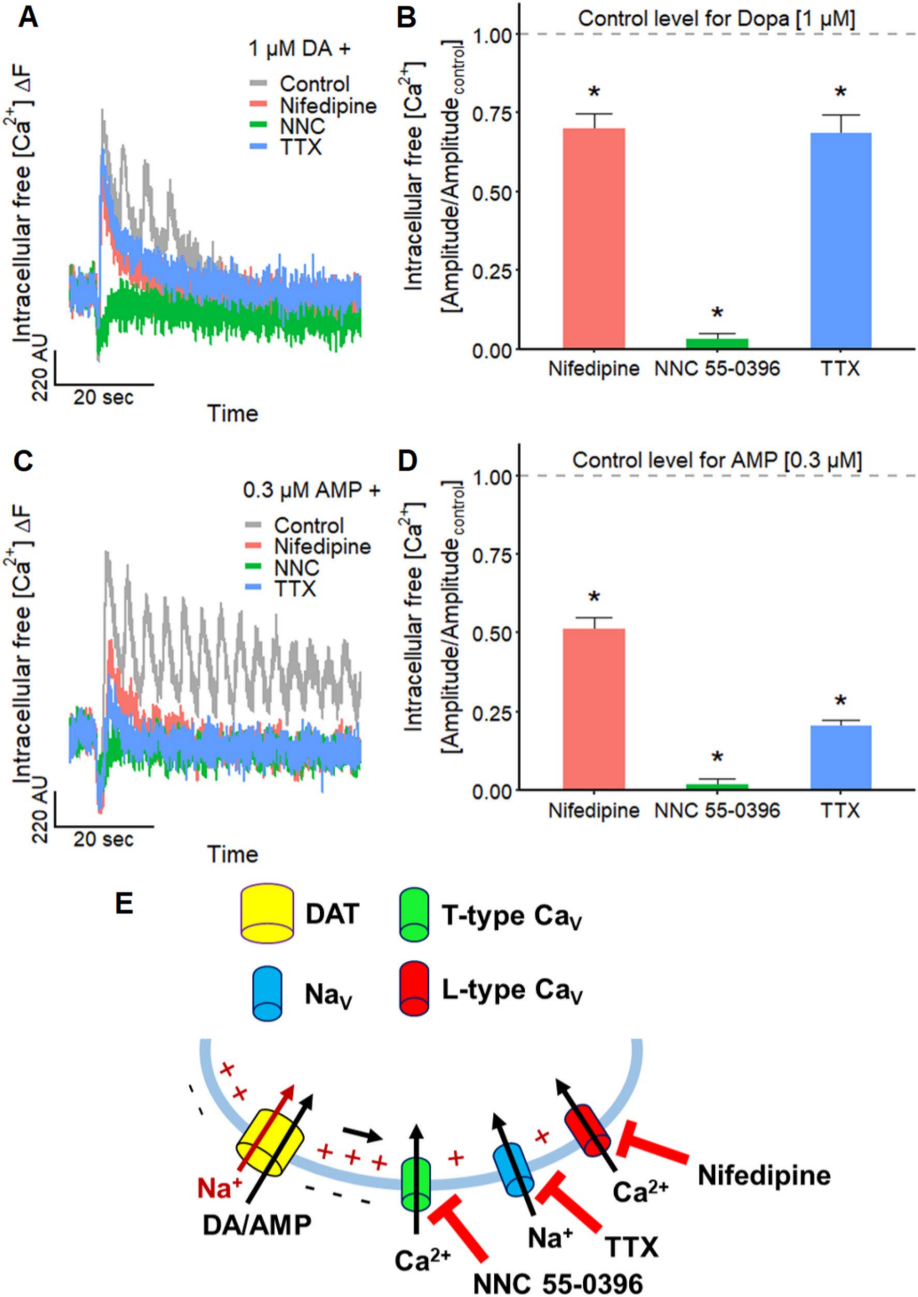
Substance-induced modulation of Ca^2+^-imaging signal oscillations. **a** Traces of a Ca^2+^-imaging experiment with LUHMES cells displaying the responses triggered by 1 µM DA during control and in the presence of 10 µM nifedipine (L-type Ca_V_ channel inhibitor), 30 µM NNC 55–0396 (NNC; T-type Ca_V_ channel inhibitor) and TTX (1 µM). Note the Ca^2+^-imaging signal oscillations during control. **b** Corresponding mean inhibitory effects of the three antagonists on the signal evoked by 1 µM DA. The amplitude was reduced compared to control (*n* = 5) to 70.2 ± 0.05% by nifedipine (*n* = 6), to 3.0 ± 0.02% by NNC 55–0396 (*n* = 6) and to 68.6 ± 0.06% by TTX (*n* = 6). Statistical significance was determined against negative control recordings (*, significant). **c** Ca^2+^-imaging traces showing the effect of 10 µM nifedipine, 30 µM NNC 55–0396 (NNC) and 1 µM TTX on the responses of the LUHMES neurons stimulated by the addition of 0.3 µM AMP. Note the Ca^2+^-imaging signal oscillations during control. **d** Mean inhibitory effects of the antagonists on the response triggered by 0.3 µM AMP. The amplitude was reduced compared to control (*n* = 6) to 51.4 ± 0.03% by nifedipine (*n* = 6), to 2.1 ± 0.02% by NNC 55–0396 (*n* = 5) and to 20.5 ± 0.02% by TTX (*n* = 6). Statistical significance was determined against negative control recordings (*, significant). **e** Schematic illustration of the results and the underlying context. The transport of DA and AMP by the DAT results in a net influx of one positive charge (Na^+^) into the cell which can activate voltage-gated ion channels via a depolarization of the membrane potential (Fig. 5)

First, we examined the involvement of Na_V_ channels in Ca^2+^-oscillations by using TTX to block action potential generation and propagation along the neurites. The signal amplitudes induced by DA (1 µM) and AMP (0.3 µM) were significantly reduced to 69 and 21%, respectively (Fig. 8a–d). Although these effects indicate a contribution of Na_V_ channels, they also suggest that there are additional components mandatory for the observed oscillations. We therefore examined the participation of L-type Ca_V_ channels in Ca^2+^-oscillations: the selective L-type Ca_V_ channel antagonist nifedipine (Helton et al. 2005) reduced the oscillation amplitude to 70% for DA and to 51% for AMP. These findings suggest that functional L-type Ca_V_ channels are involved in Ca^2+^-oscillations. Their presence is in line with the expression levels of L-type Ca_V_ channel mRNA (Fig. S2), indicating a high expression of Ca_V_1.2. As the presence of functional T-type Ca_V_ channels is supported by high mRNA levels of Ca_V_3.2 (Fig. S2), we investigated the impact of T-type Ca_V_ channels on the DA/AMP induced oscillations: the selective T-type Ca_V_ channel blocker NNC 55–0396 (NNC, Huang et al. 2004) caused a strong reduction of the amplitude to 2–3% (> 95% inhibition) (Fig. 8a–d). This indicates a major contribution of T-type Ca_V_ channels in Ca^2+^-oscillations.

One may ask why T-type Ca_V_ channels take such an important role here (Fig. 8e): These Ca_V_ channels have a lower activation threshold compared to L-type Ca_V_ channels (Helton et al. 2005; Lieb et al. 2014). This higher voltage sensitivity enables a stronger electrical coupling with the DAT (Cameron et al. 2015), but it may also have important pathophysiological (e.g., epilepsy (Huc et al. 2009; Cain and Snutch 2013)) and toxicological implications.

In summary, this final set of experiments showed that LUHMES cultures, assessed by whole-well Ca^2+^-imaging as an end point not only can be used to investigate modulations of ion channels, neurotransmitter receptors, and transporters that affect individual neurons, but also for identification of chemicals that alter synchronous activity in this test system.

## Conclusions and outlook

LUHMES have in the past been used as toxicity test system with biochemical and morphological end points (Scholz et al. 2011; Smirnova et al. 2016; Delp et al. 2018b, 2019; Brüll et al. 2020). To the best of our knowledge, we provide here for the first time an extensive overview of neurophysiological changes triggered by external chemicals in LUHMES neurons. We show a broad panel of such responses to exemplify the functioning and performance of LUHMES as test system of functional neurotoxicity. Some of the results yield further neurobiological characterization of the test system.

For instance, our results indicate the functional expression of P2X3 receptors, as demonstrated by the inhibitory effect of A-317491 on the response evoked by α,β-meATP. We also illustrated the high reproducibility of the differentiation of LUHMES neurons by the low standard deviation of 0.16 of five pEC_50_ values (mean of 7.52) determined for the responses of five differentiations to α,β-meATP. A use case was given by the characterization of suramin and by showing how the system can be used to provide exact quantitative data on agonist and antagonist potencies and specificities. In the future, the identification of side effects of antiepileptic and anti-inflammatory drugs addressing purinergic receptors could be of interest, due to their wide distribution in the nervous system (Burnstock and Verkhratsky 2012; Di Virgilio and Vuerich 2015; Riquelme et al. 2020).

In a further step, we showed that high-throughput Ca^2+^ assays can substitute patch clamp for many applications, and provide a central toxicological platform to investigate diverse neurofunctional modulators/toxicants. This method enables the utilization of adherent cells in an intact neuronal network compared to automated patch clamp, where cells need to be detached. This assay served, e.g., as a useful tool for the detection of marine neurotoxins such as TTX, STX and CTX. It may be used in the future also for, e.g., cyanobacterial toxins, like kalkitoxin (LePage et al. 2005). The possibility of examining use-dependent effects on this Na_V_ channel is also meaningful for the research of anticonvulsants, like phenytoin (Goldenberg 2010; Brodie 2017), and the detection of side effects of local anesthetics or pyrethroids on CNS Na_V_ channels (Groban 2003; Mather et al. 2005; Neal et al. 2010; Cao et al. 2011; Casida and Durkin 2013).

Scholz et al. (2011) mentioned the presence of TTX-sensitive Na_V_ channels in this cell model. We went further by identifying Na_V_1.2 as the major functionally active sodium channel (Fig. 4f). By establishing a procedure to utilize the LUHMES neurons in automated patch clamp, we overcame the low throughput of manual patch clamp.

A major outcome of our study, besides the broad test system description as necessary basis for further work, was the demonstration that LUHMES neurons are functionally coupled over long distances (entire well). The finding of oscillations of activity was very clear for different stimuli such as DA, AMP and CTX, and we provided a description of the robustness of the phenomenon. A thorough investigation of the underlying biology was out of the scope of this study, but it is an important goal for the future. To substantiate our findings, and to ensure they are not strange random observations, we provided some mechanistic links: DAT and T-type Ca_V_ were found to be major players in such culture [Ca^2+^]_i_ oscillations, and also Na_V_ contributed to them. Potential applications for this assay could be the identification of all agents that disturb neuronal network functions, such as antipsychotics, seizurogenic substances and antiepileptic drugs that do not solely affect the GABA-glutamate system.

In summary, we highlighted the suitability of LUHMES neuronal cultures as powerful tool for high-throughput neuronal toxicity screening using industry-applicable automated patch clamp and Ca^2+^-imaging. Furthermore, we revealed the presence of several meaningful targets on the LUHMES neurons for the assessment of neurotoxicity and exemplified this in several case studies. In future studies, it would be worthwhile investigating the effects of the tested neurotoxicants on 3D models and co-culture systems with astrocytes (Brüll et al. 2020).

## Electronic supplementary material

Below is the link to the electronic supplementary material.

Supplementary file1 (DOCX 2050 KB)

## Abbreviations

AMP: Amphetamine
ATP: Adenosine 5'-triphosphate
cAMP: N6,2′-0-dibutyryl 3′,5′-cyclic adenosine monophosphate
Ca_v_: Voltage-gated calcium channel
DA: Dopamine
DAT: Dopamine transporter
K_v_: Voltage-gated potassium channel
LUHMES: Lund human mesencephalic
MEA: Microelectrode array
Na_v_: Voltage-gated sodium channel
P2X: Ionotropic purinergic
P2Y: Metabotropic purinergic
pCTX: Pacific ciguatoxin
pEC_50_: Negative logarithm of the half-maximal effective concentration
pIC_50_: Negative logarithm of the half-maximal inhibitory concentration
PLO: Poly-l-ornithine
STX: Saxitoxin
TEA: Tetraethylammonium
TTX: Tetrodotoxin
VTD: Veratridine
