# Supplementary material for: Human neuronal signaling and communication assays to assess functional neurotoxicity
Source: Arch Toxicol. 2020 Dec 2;95(1):229–52. doi: 10.1007/s00204-020-02956-3 (PMC7811517; doi:10.1007/s00204-020-02956-3)
Supplement: Supplementary file 1 — Supplementary file1 (DOCX 2050 KB) [file 204_2020_2956_MOESM1_ESM.docx]

**Supplementary information for**

**Human neuronal signaling and communication assays to assess functional neurotoxicity**

*Dominik Loser^1,2,4^, Jasmin Schaefer^1,2^, Timm Danker^2^, Clemens Möller^4^, Markus Brüll^3^, Ilinca Suciu^3^, Anna-Katharina Ückert^3^, Stefanie Klima^3^, Marcel Leist^3,#^ , Udo Kraushaar^1,#^*

^1^ NMI Natural and Medical Sciences Institute at the University of Tuebingen, 72770 Reutlingen, Germany

^2^ NMI TT GmbH, 72770 Reutlingen, Germany

^3^ In vitro Toxicology and Biomedicine, Dept. inaugurated by the Doerenkamp-Zbinden foundation, University of Konstanz, 78457 Konstanz, Germany

^4^ Life Sciences Faculty, Albstadt-Sigmaringen University, 72488 Sigmaringen, Germany

^#^ these authors contributed equally

| **Table of Contents** | | |
| --- | --- | --- |
| **Fig. S1** | Functional P2X receptor expression in LUHMES neurons. | page 2 |
| **Fig. S2** | Gene expression profile of LUHMES cells. | page 3 |
| **Fig. S3** | Effect of P2X receptor agonist α,β-meATP on LUHMES neurons. | page 4-5 |
| **Fig. S4** | Effects of marine biotoxins on LUHMES neurons. | page 5-6 |
| **Fig. S5** | Firing behavior and biophysical properties of Na_V_ channel currents of LUHMES neurons. | page 7-8 |
| **Fig. S6** | Inhibition of dopamine transporter (DAT) activity in LUHMES cells. | page 8 |
| **Table S1** | Compound list. | page 9 |
| **Table S2** | Overview of pEC_50_ values for agonist experiments. | page 10 |
| **Table S3** | Overview of pIC_50_ values for antagonist experiments. | page 11 |
| **Table S4** | Overview of concentrations and replicates. | page 12-13 |


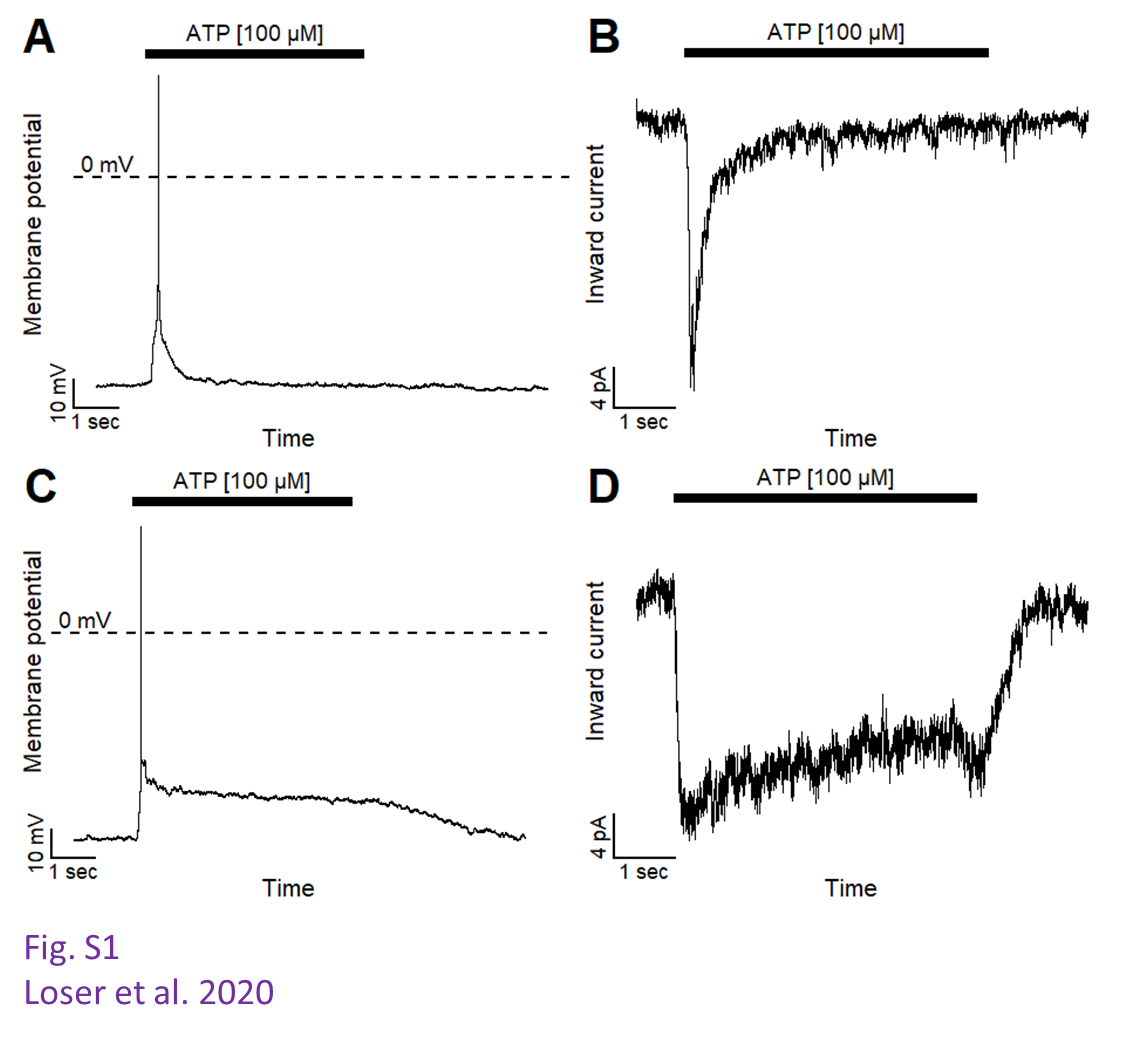


**Fig. S1. Functional P2X receptor expression in LUHMES neurons.**

Manual patch clamp recordings of the responses of LUHMES neurons evoked by the application of 100 µM ATP for 5 sec. (A) Action potential firing in current-clamp recording with a short depolarization (n = 7). (B) Voltage-clamp recording with a fast inactivating inward current (n = 15). Besides the fast kinetics, (C) a slow repolarization of the membrane potential was also recorded in current-clamp (n = 4). (D) This finding was substantiated by voltage-clamp recordings that showed slowly inactivating inward currents (n = 13).


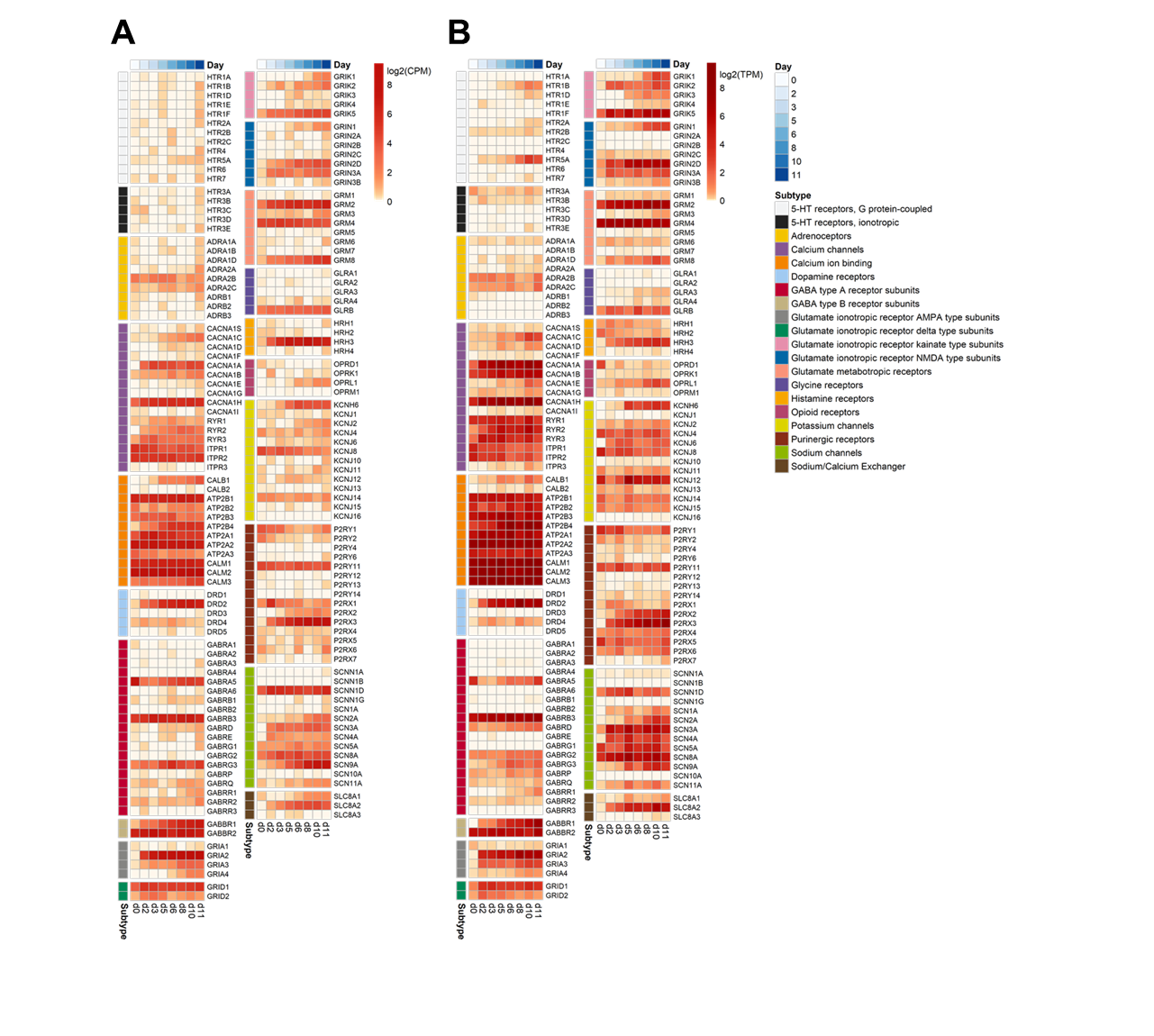


**Fig. S2. Gene expression profile of LUHMES cells.**

Five biological replicates were generated from LUHMES cells differentiated for 2, 3, 5, 6, 8, 10, and 11 days, as well as from undifferentiated LUHMES cells (day 0). The heatmaps visualize the normalized and logarithmic counts for each gene (rows) of neurotransmitter receptors, ion channels, and calcium binding proteins, and day of differentiation (columns, indicated in blues). Darker red indicates high expression, orange indicates low expression, and white indicates no expression. The genes are clustered by the gene group (e.g. receptor or ion channel subtype). Gene groups are indicated by color in the first column. Samples were analyzed via (A) the TempO-Seq assay and via (B) traditional whole genome RNA-sequencing (RNAseq).


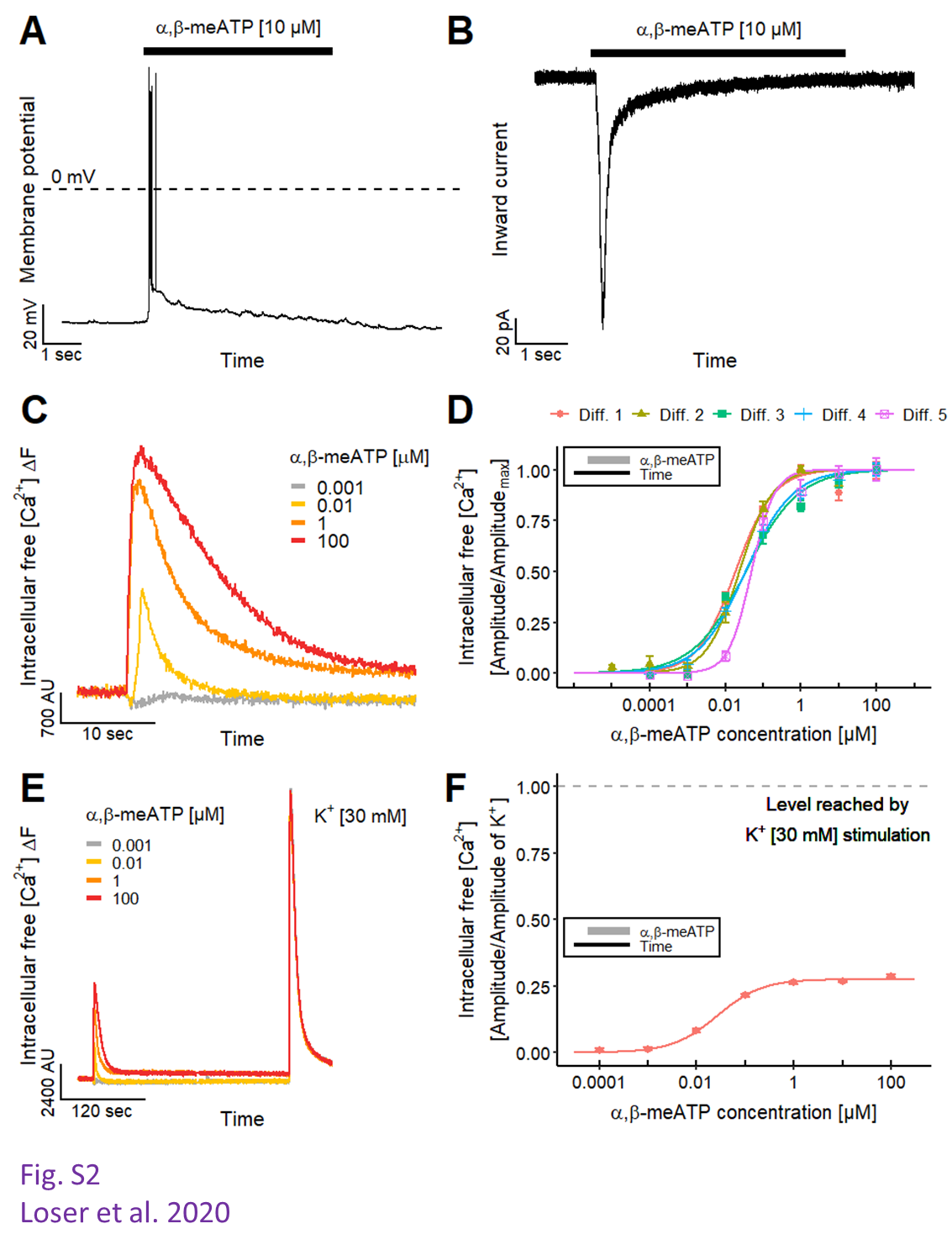


**Fig. S3. Effect of P2X receptor agonist α,β-meATP on LUHMES neurons.**

(A-B) Traces of manual patch clamp recordings of the application of 10 µM α,β-meATP for 5 sec. (A) In current clamp, the cells responded either with tonic (n = 21) or phasic firing behavior (n = 25, not shown) due to a transient depolarization of the membrane potential. (B) In voltage clamp, an inward current with fast activation and inactivation kinetics was measured in all recorded cells (n = 16). (C) Traces of Ca^2+^-imaging signals produced by the addition of different concentrations of α,β-meATP on LUHMES neurons. (D) Comparison of concentration-response curves recorded from five differentiations (Diff. 1-5) for the effect of α,β-meATP. The resulting pEC_50_ values show a very low variability between the differentiations. (E) Exemplary traces of Ca^2+^-imaging displaying the responses evoked by different concentrations of α,β-meATP (1^st^ peak) followed by the second application of 30 mM K^+^ (2^nd^ peak). (F) The concentration-response curve showing the mean over 5 differentiations, for which the amplitude evoked by α,β-meATP was normalized to the corresponding amplitude of the K^+^-application. The maximal amplitude of α,β-meATP reached 27.6% of the K^+^ amplitude with a pEC_50_ value of 7.58 ± 0.04. Note the treatment schemes, illustrating the experimental design. Detailed data on n numbers are found in table S4.


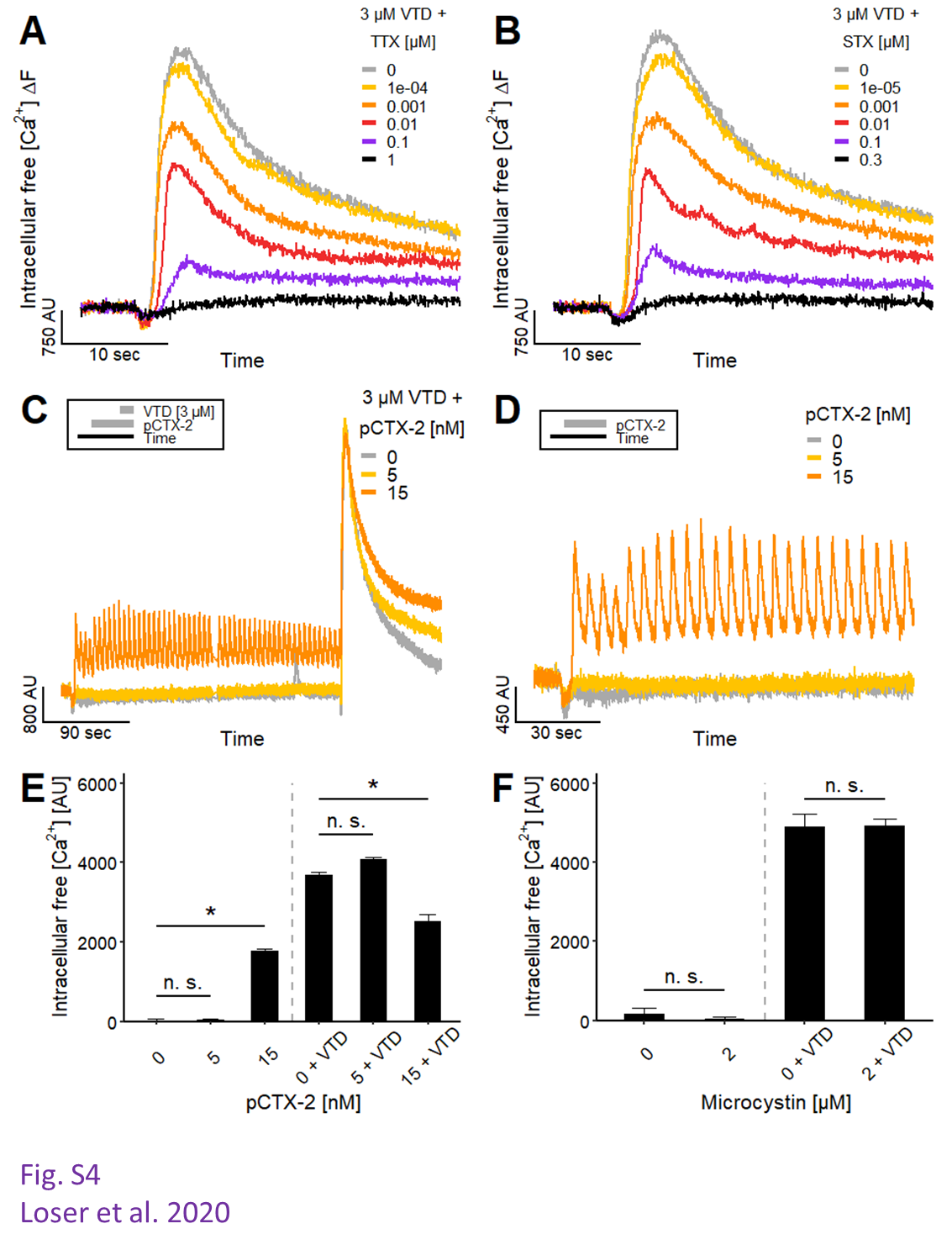


**Fig. S4. Effects of marine biotoxins on LUHMES neurons.**

(A-B) Traces of Ca^2+^-imaging experiments showing the responses of LUHMES cells evoked by the application of 3 µM VTD in the presence of different concentrations of (A) TTX and (B) saxitoxin (STX). (C-D) Ca^2+^-imaging signals displaying the effect of (C) pacific ciguatoxin-2 (pCTX-2), a Na_V_ channel modulator, followed by the application of 3 µM VTD on LUHMES neurons. 15 nM pCTX-2 induced an increase of the baseline and oscillations of the Ca^2+^-imaging signal. (D) Magnification of the effects of pCTX-2. Note the oscillations triggered by 15 nM pCTX-2. (E) The response evoked by 15 nM pCTX-2 (n = 6) reached an amplitude of 1779 ± 50 AU. At 5 nM pCTX-2 (n = 4) no effect was detected compared to control (n = 6). The amplitude induced by 3 µM VTD, after the addition of pCTX-2, was significantly reduced by 15 nM pCTX-2 taking the baseline increase into account. Otherwise the effect of 15 nM pCTX-2 on the VTD response would not be significant. (F) The biotoxin microcystin-LF was tested in a concentration of 2 µM followed by the application of 3 µM VTD. The results show no significate effect of 2 µM microcystin-LF (n = 6) compared to control (n = 5), as well as on the response triggered by 3 µM VTD. Note the treatment scheme (upper left corner), illustrating the experimental design. (E-F) Statistical significance was determined against negative control recordings (*, significant; n. s., not significant).


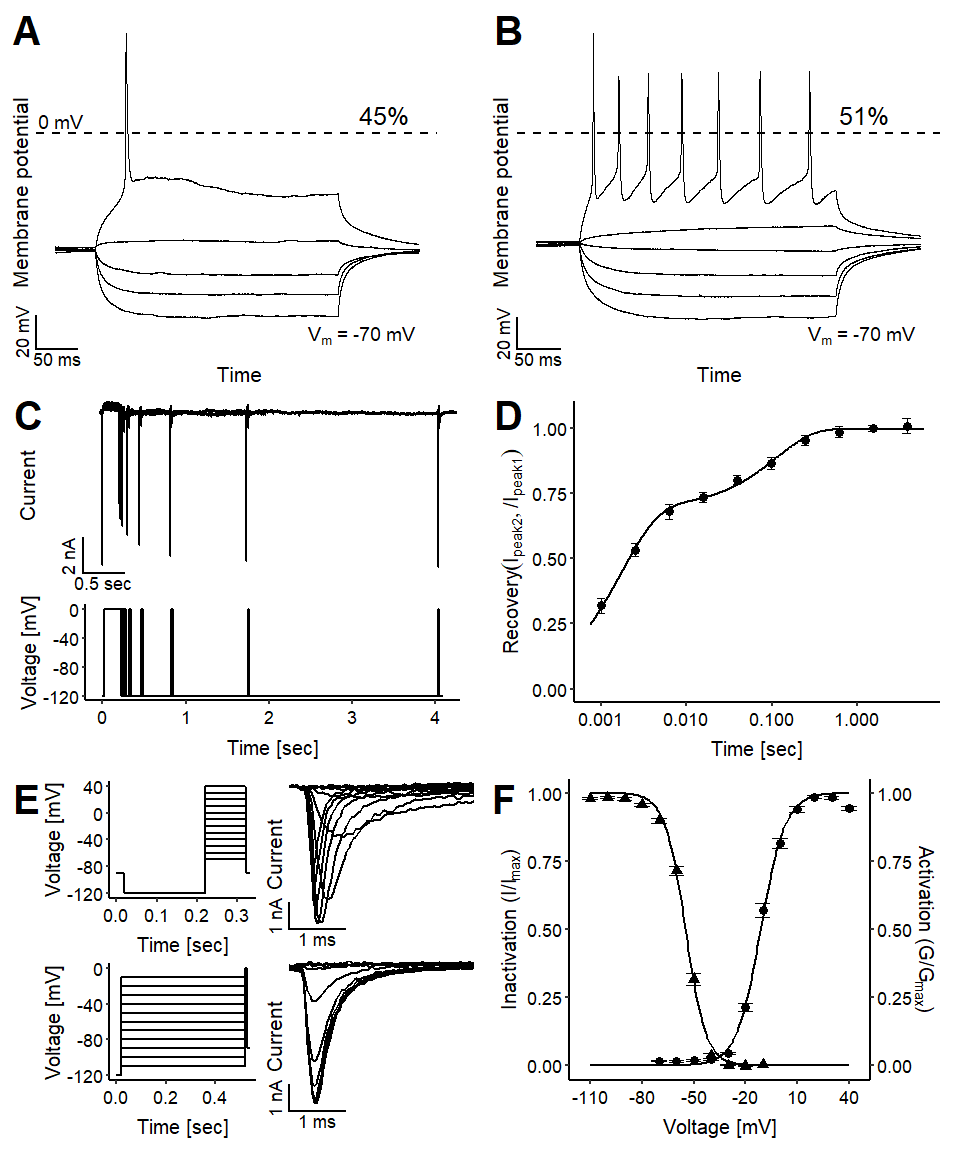


**Fig. S5. Firing behavior and biophysical properties of Na_V_ channel currents of LUHMES neurons.**

(A-B) Investigation of the firing behavior of LUHMES neurons using manual patch clamp. The cells were stimulated with 300 ms hyper- and depolarizing current pulses from holding potential (V_m_; n = 274). (A) 45% of the cells showed phasic and (B) 51% tonic firing behavior. Depolarization failed to induce action potentials in only 4% of the cells. (C-F) Automated patch clamp recordings of voltage-gated sodium (Na_V_) channels expressed in LUHMES neurons. (C) Exemplary traces of the Na^+^ current triggered by the pulse protocol illustrated below. (D) The recovery from steady-state inactivation of the Na_V_ channels was fitted biexponentially, resulting in a slow component with A_1_ = 0.3 and τ_1_ = 112.5 ms and a fast component with A_2_ = 0.7 and τ_2_ = 1.7 ms (n = 6). (E) On the left side, pulse protocols for the investigation of the activation (top) and the steady-state inactivation (bottom) kinetics of the Na_V_ channel currents are presented. On the right side, the corresponding exemplary traces of the Na^+^ currents evoked by the test pulses are displayed. (F) Mean voltage-dependent conductance (G/G_max_) for the activation kinetics (●) with a half maximal activation voltage (V_50_) of -11.3 mV and a constant (k) of 7.0 (n = 13) and steady-state inactivation (I/I_max_) (▲) with V_50_ = -54.9 mV and k = -5.9 (n = 21). The curves were fitted with the Boltzmann equation.


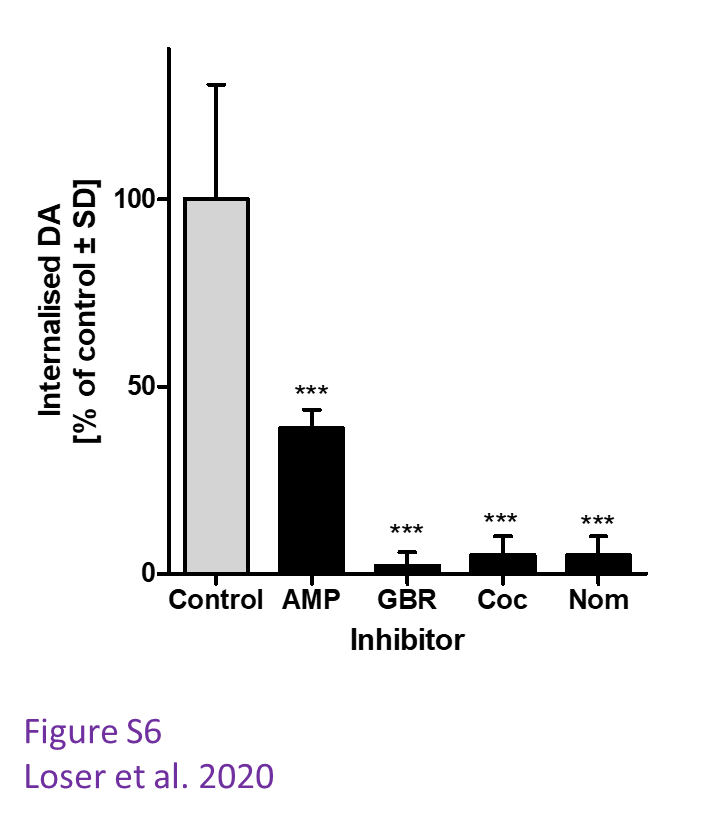


**Fig. S6. Inhibition of dopamine transporter (DAT) activity in LUHMES cells.**

The uptake of radioactively-labelled DA ([^3^H]DA) by LUHMES cells was inhibited by the DAT antagonists amphetamine (AMP, 10 µM), GBR12935 (GBR, 1 µM), cocaine (Coc, 100 µM) and nomifensine (Nom, 40 µM). All data are means ± SD of triplicate data. ***p<0.0001 according to ANOVA, followed by Dunnett’s post-hoc test. Parallel time course measurements showed that at 10 minutes, 50% of the maximum uptake had occurred. Altogether, cells accumulated about 6% of all DA in the culture medium. Inhibition studies performed for 30 and 60 min (not shown) yielded essentially similar data.

**Table S1. Compound list.**

**Table S2. Overview of pEC_50_ values for agonist experiments.**

**Table S3. Overview of pIC_50_ values for antagonist experiments.**

**Table S4. Overview of concentrations and replicates.**

(Table S4 continues on the next page.)

**Table S4. Overview of concentrations and replicates. (continued)**
